# Correction: TAC102 Is a Novel Component of the Mitochondrial Genome Segregation Machinery in Trypanosomes

**DOI:** 10.1371/journal.ppat.1005750

**Published:** 2016-07-01

**Authors:** Roman Trikin, Nicholas Doiron, Anneliese Hoffmann, Beat Haenni, Martin Jakob, Achim Schnaufer, Bernd Schimanski, Benoît Zuber, Torsten Ochsenreiter

## Notice of Republication

This article was republished on June 16, 2016, to correct errors in the figure legends that were introduced during the typesetting process. Please download this article again to view the correct version. The originally published, uncorrected article and the republished, corrected article are provided here for reference.

## Supporting Information

S1 File Originally published, uncorrected article. (PDF)

S2 File Republished, corrected article. (PDF)
