# Supplementary figures and images for: TAC102 Is a Novel Component of the Mitochondrial Genome Segregation Machinery in Trypanosomes
Source: PLoS Pathog. 2016 May 11;12(5):e1005586. doi: 10.1371/journal.ppat.1005586 (PMC4864229; doi:10.1371/journal.ppat.1005586)

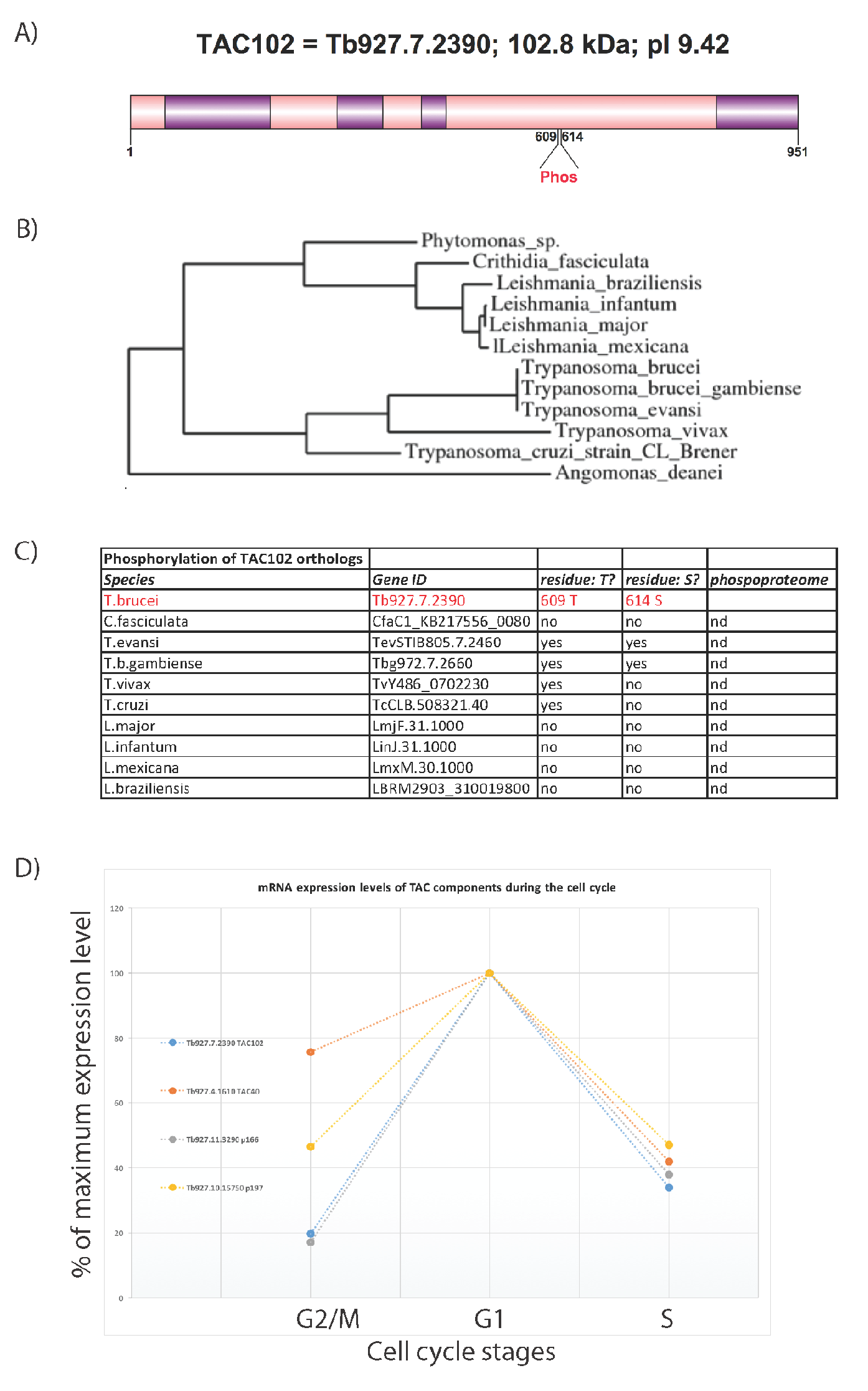

Supplement: S1 Fig — A–a stick figure displaying conserved regions (violet) of the TAC102 protein sequence as well as phosphorylation sites at positions 609 and 614. The non-conserved regions are depicted in pink. B–a phylogenetic tree showing conservation of TAC102 among Kinetoplastea. The tree was reconstructed using PhyML based on a manually curated sequence alignment using MUSCLE. C–a table showing conservation of the two identified phosphorylation sites (609T and 614S) in TAC102 orthologs in several Kinetoplastea. D–mRNA expression levels during the G1, S and G2/M phases of the cell cycle of PCF trypanosomes. Shown are the relative expression levels normalized to the highest expression of each of the transcripts (100%). The data is based on cells sorted by DNA content followed by mRNA extraction and spliced leader based Illumina sequencing as described previously [58]. Shown are the examples of the currently known TAC components. (TIFF) [file ppat.1005586.s001.tiff]

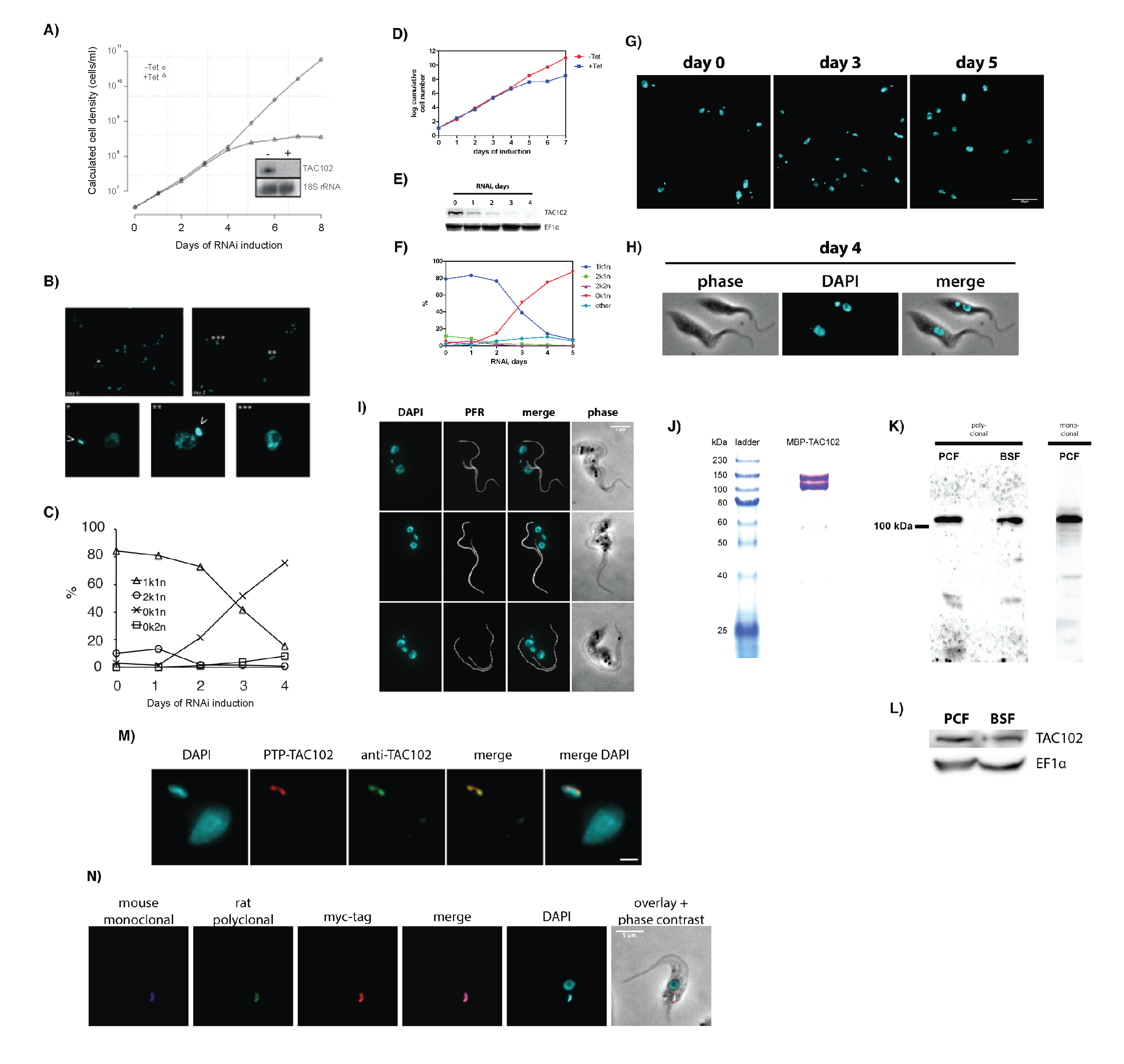

Supplement: S2 Fig — A-C: RNAi against the ORF of TAC102 in PCF cells. A–a growth curve showing the onset of a growth defect after day 4 of RNAi induction. Inset: a northern blot confirming downregulation of TAC102 mRNA after two days of RNAi induction. 18S rRNA is used as a loading control. B–epifluorescence images (DAPI staining) showing missegregation and loss of kDNA after two days of RNAi induction. Comparison of a cell with a “normal” kDNA (*), with a large kDNA (**) and without kDNA (***). C–percentage of cells with different k-n-combinations within the course of TAC102 RNAi. The number of 1k1n cells (triangles) decreases significantly and 0k1n cells (crosses) become the dominant cell type. D-I: RNAi against the 3’-UTR of TAC102 in PCF cells. D–a growth curve showing the onset of a growth defect after day 4 of RNAi induction. E–a western blot showing a decrease in the amount of TAC102 protein upon its depletion by RNAi. EF1α used as a loading control. F–percentage of cells with different k-n-combinations within the course of TAC102 RNAi. The number of 1k1n cells (blue circles) decreases significantly and 0k1n cells (red triangles) become the dominant cell type. G–epifluorescence images (DAPI staining) showing loss of kDNA after three and five days of RNAi induction. H–epifluorescence images showing an example of cells with missegregated kDNA on day 4 of RNAi induction, one with a small kDNA and another with a big one. I–fluorescence images showing examples of induced cells (3 days of RNAi) that have lost or missegregated the kDNA. DNA is stained with DAPI (cyan) and flagella are stained with anti-PFR antibody (gray). J-N: recombinant TAC102 and antibodies against TAC102. J–a Coomassie stained SDS-PAAG showing expression of the recombinant version of TAC102 with MBP at its N-terminus in E. coli. After purification on amylose two major forms of the recombinant protein are detected, the bigger one (full-length) and the smaller one (C-terminally processed by bacteria). K–western blot [file ppat.1005586.s002.tiff]

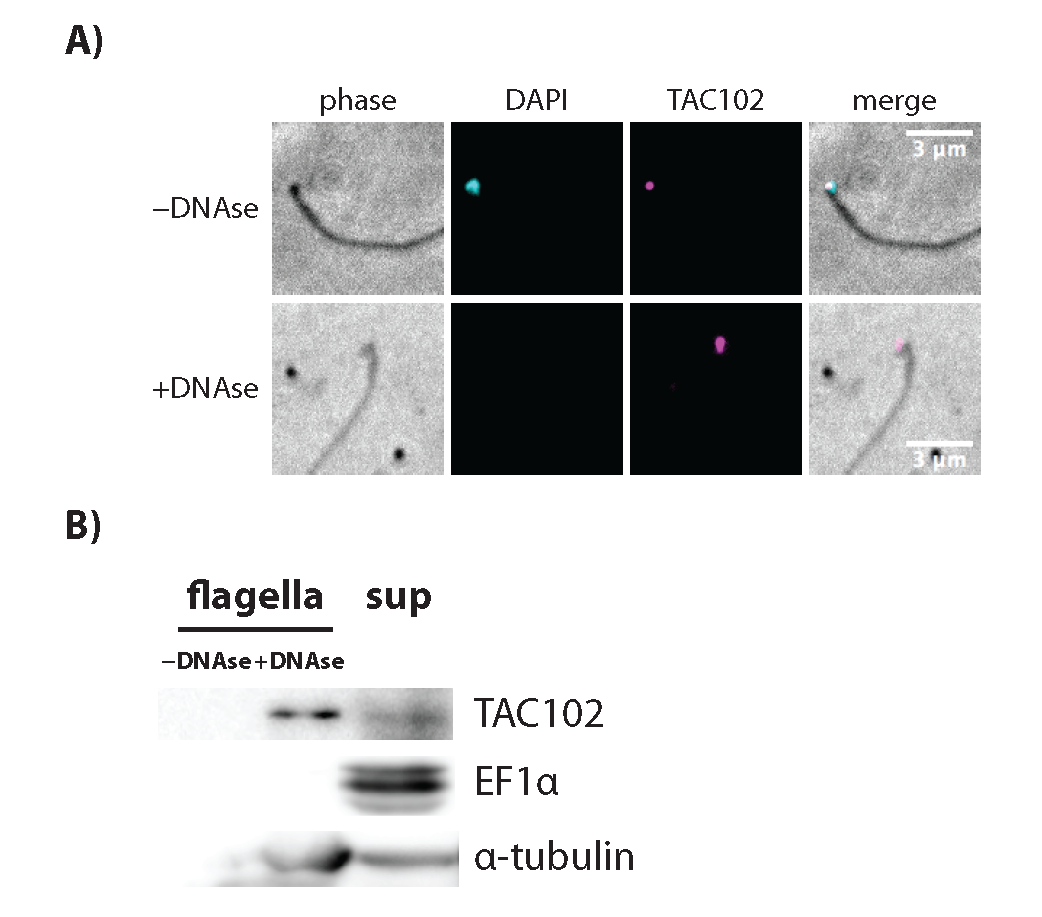

Supplement: S3 Fig — Flagella were extracted from PCF trypanosomes with 0.5% TritonX-100, as described in Materials and Methods, and treated with DNAse I or left untreated. A–immunofluorescence images showing: an untreated flagellum (upper panel) that retains the kDNA (stained with DAPI, cyan) and TAC102 (magenta); a DNAse I–treated flagellum (lower panel) that has lost the kDNA but retains TAC102. B–a western blot showing that TAC102 is present in both the flagellar extract and the supernatant. The same is observed for α-tubulin. EF1α a cytosolic protein, is found only in the soluble fraction. Since flagella that were not treated with DNAse I were difficult to handle we could not detect any of these proteins in that fraction. For each flagellar fraction, the loaded cell equivalent was twice more than that of the supernatant. (TIFF) [file ppat.1005586.s003.tiff]

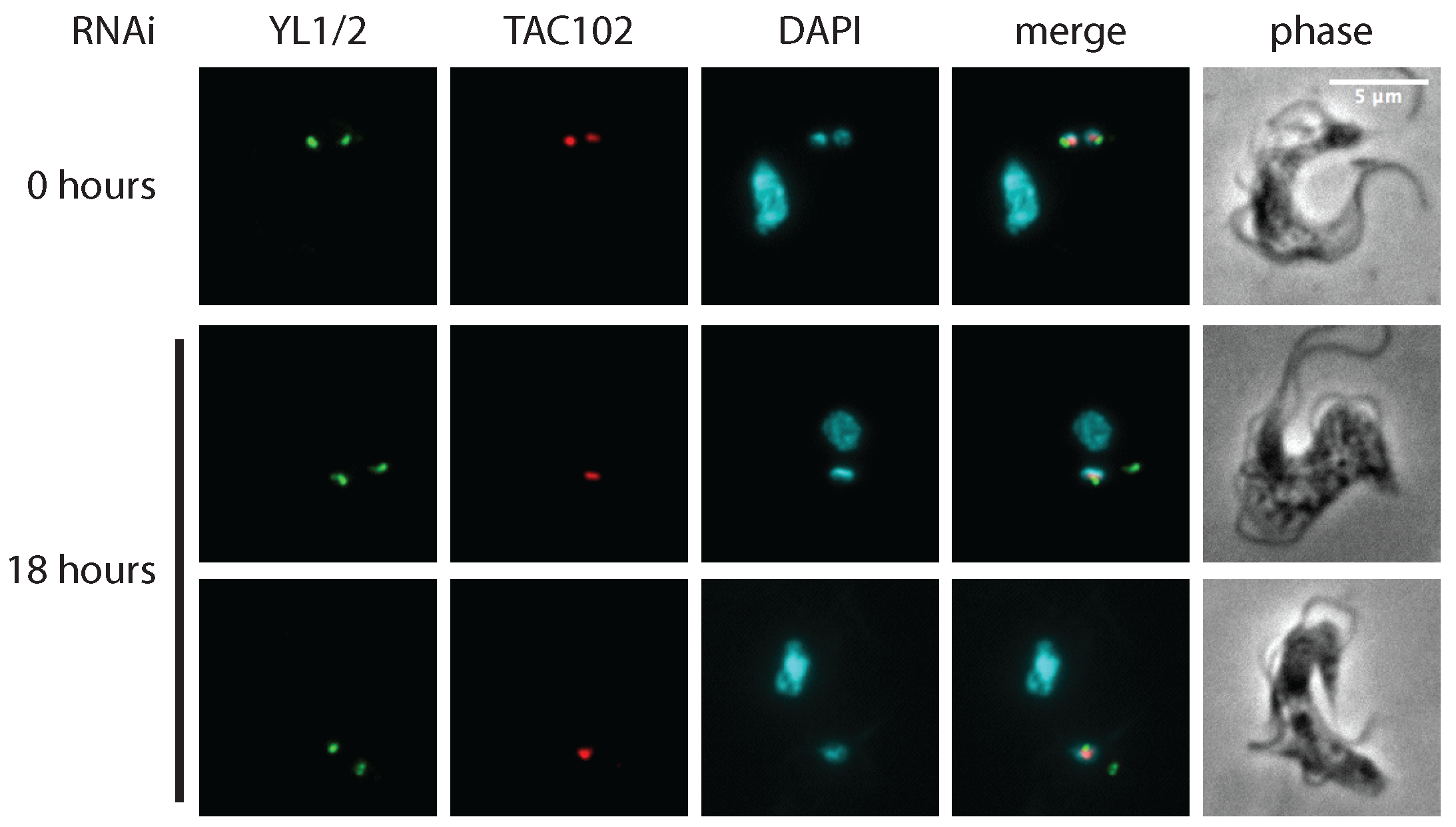

Supplement: S4 Fig — At this early time point, some cells lose the TAC102 signal as well as the kDNA, but it happens preferably at the more posterior basal body (examples in the middle panel and the lower panel, compare to non-induced cells in the upper panel). YL1/2 is used as a basal body marker (green), TAC102 is shown in red, DAPI staining of DNA in cyan. (TIFF) [file ppat.1005586.s004.tiff]

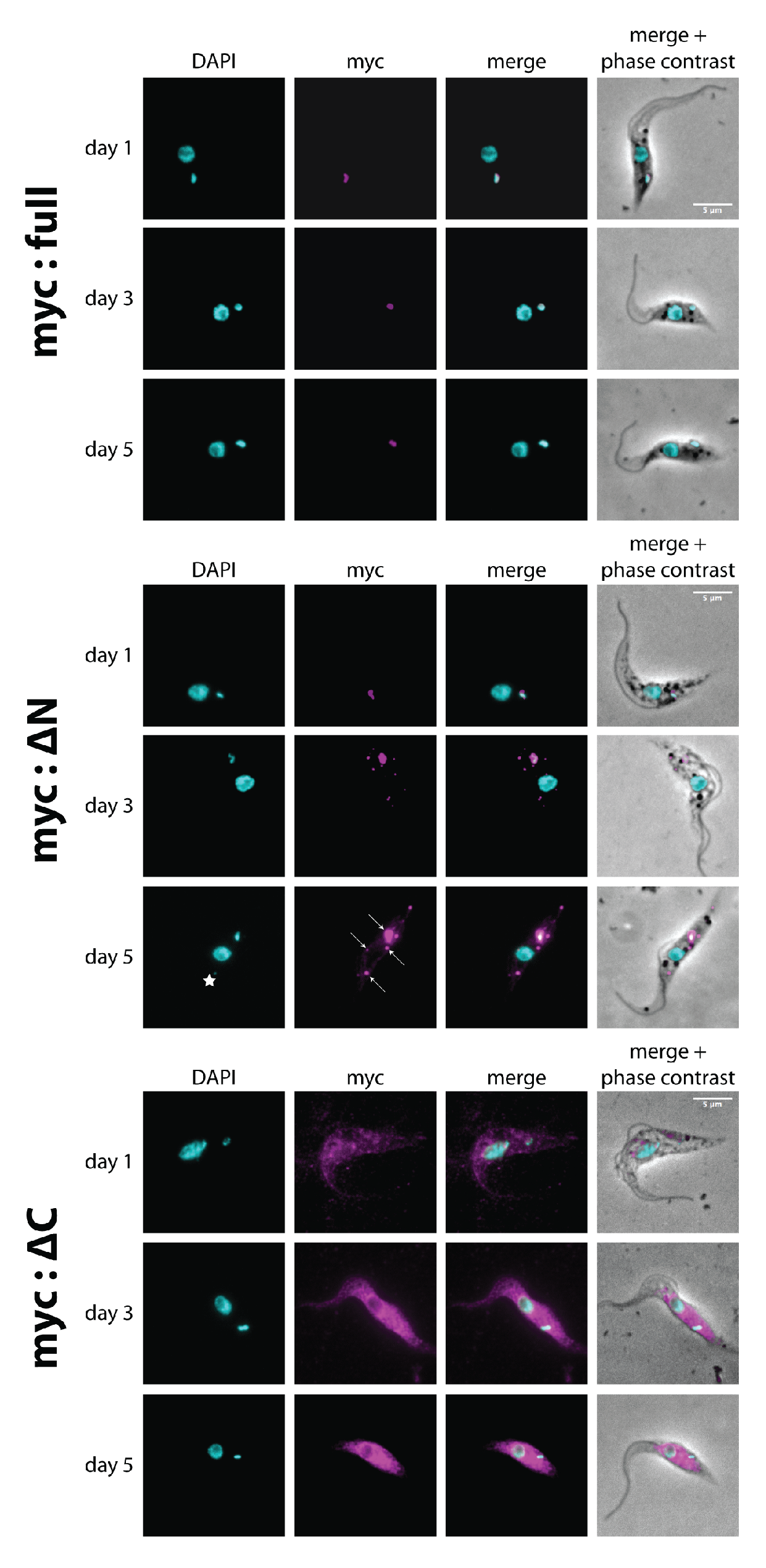

Supplement: S5 Fig — DNA is stained with DAPI (cyan) and myc-tagged proteins (visualized by anti-myc antibody) are shown in magenta. Expression of the myc:full protein (upper set of panels) does not affect the kDNA and the protein localizes to the position of the endogenous TAC102. Expression of the myc:ΔN protein (middle set of panels) causes appearance of ancillary kinetoplasts (day 5, indicated with a star); the protein accumulates in multiple locations (day 5, indicated with arrows) and is present at the site of the ancillary kinetoplast. Expression of the myc:ΔC protein (lower set of panels) does not affect the kDNA and the protein localizes to the cytoplasm. (TIFF) [file ppat.1005586.s005.tiff]

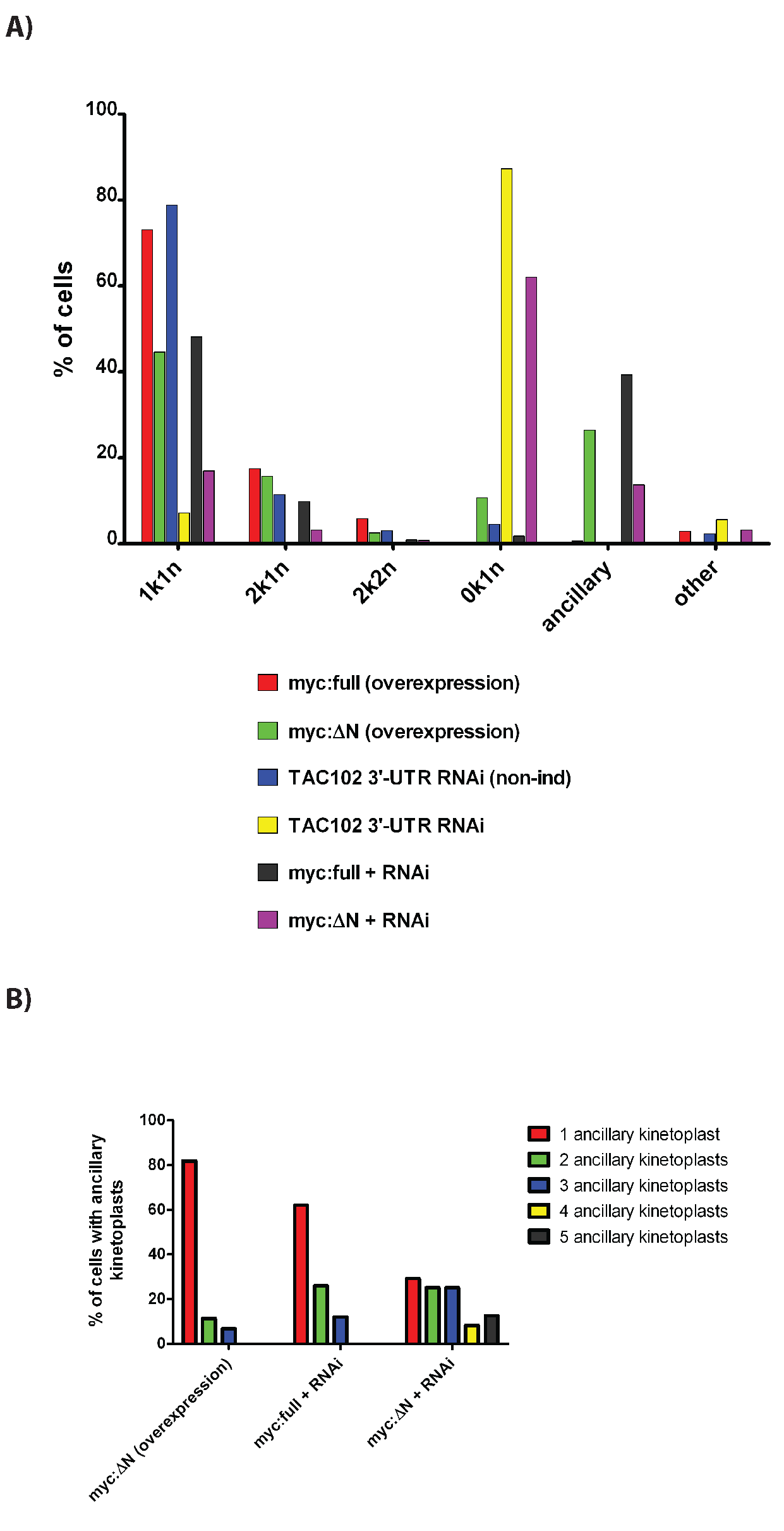

Supplement: S6 Fig — A–a column chart showing percentages of cells with different k-n-numbers in the following PCF cell lines: myc:full, overexpression for five days (red); myc:ΔN (overexpression for five days (green); RNAi against the 3’-UTR of TAC102, non-induced cells (blue); RNAi against the 3’-UTR of TAC102, induced for five days (yellow); myc:full in the background of RNAi against the 3’-UTR of TAC102, induced for five days (gray); myc:ΔN in the background of RNAi against the 3’-UTR of TAC102, induced for five days (violet). B–a column chart showing percentages of cells with different numbers of ancillary kinetoplasts per cell. The percentages displayed are of the cells with ancillary kinetoplasts, and not of all cells in the population. The data is shown for three PCF cell lines where “extra” kDNAs were observed: myc:ΔN overexpression for five days; myc:full in the background of RNAi against the 3’-UTR of TAC102, induced for five days; myc:ΔN in the background of RNAi against the 3’-UTR of TAC102, induced for five days. (TIFF) [file ppat.1005586.s006.tiff]

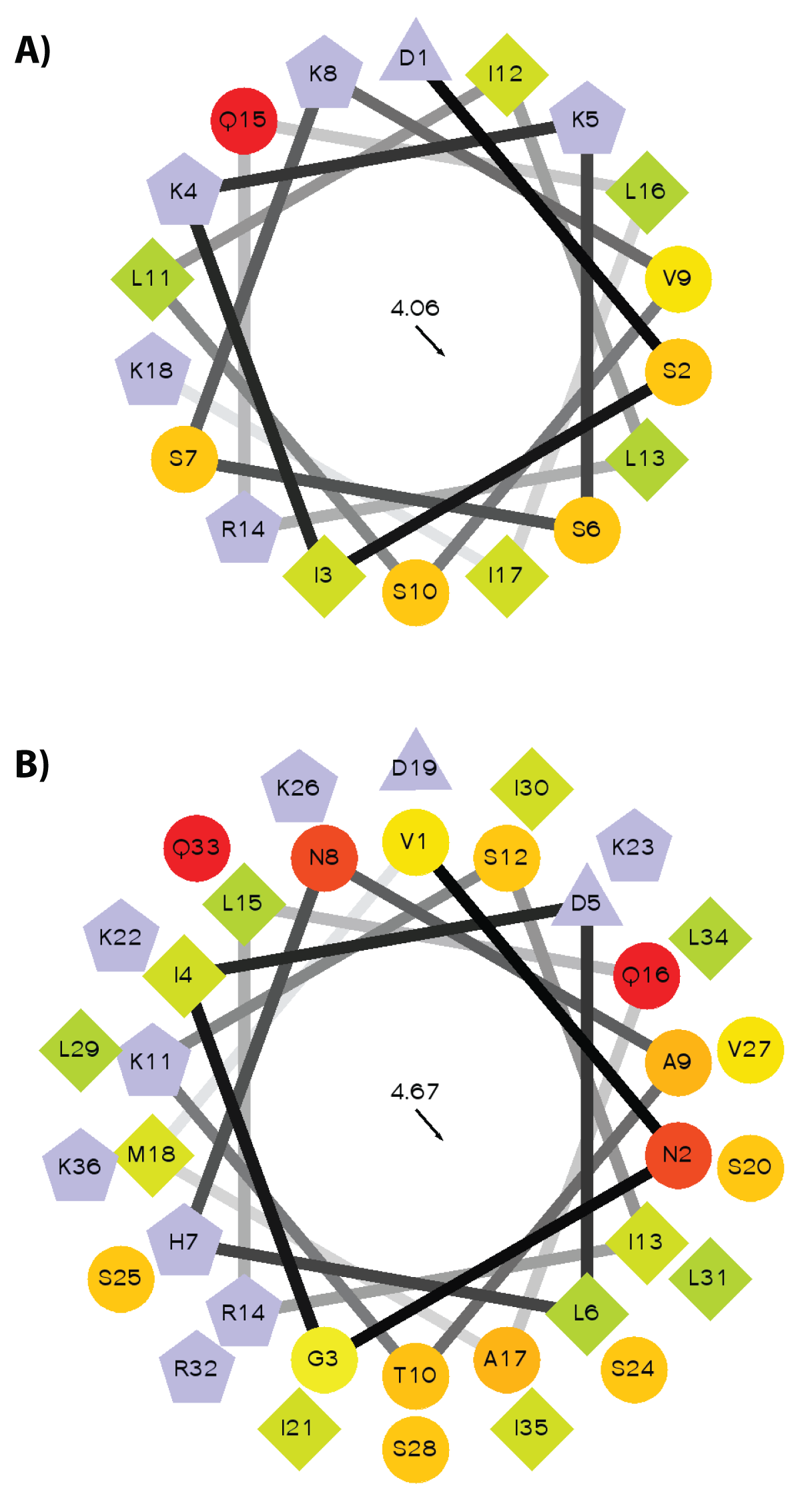

Supplement: S7 Fig — The schematic alpha-helices show the last 18 aa (A) or 36 aa (B) of the C-terminal sequence of TAC102. The schemes were constructed by the online tool available at http://rzlab.ucr.edu/scripts/wheel/wheel.cgi. Hydrophilic residues are shown as circles, hydrophobic residues–as diamonds, potentially negatively charged–as triangles, and potentially positively charged–as pentagons. Hydrophobicity has a color code: the most hydrophobic residues are green, and the amount of green decreases proportionally to the hydrophobicity, with zero hydrophobicity shown in yellow. Hydrophilic residues are red, with pure red being the most hydrophilic (uncharged) residue, and the amount of red decreasing proportionally to the hydrophilicity. The potentially charged residues are light blue. Based on the distribution of amino acid residues in these helices, both the last 18 aa (A) and the last 36 aa (B) of the C-terminal sequence of TAC102 form amphipathic helices. The last 18 aa of TAC102: DSIKKSSKVSLILRQLIK (numbers 1–18 in scheme A) The last 36 aa of TAC102: VNGIDLHNATKSIRLQAMDSIKKSSKVSLILRQLIK (numbers 1–36 in scheme B) (TIFF) [file ppat.1005586.s007.tiff]

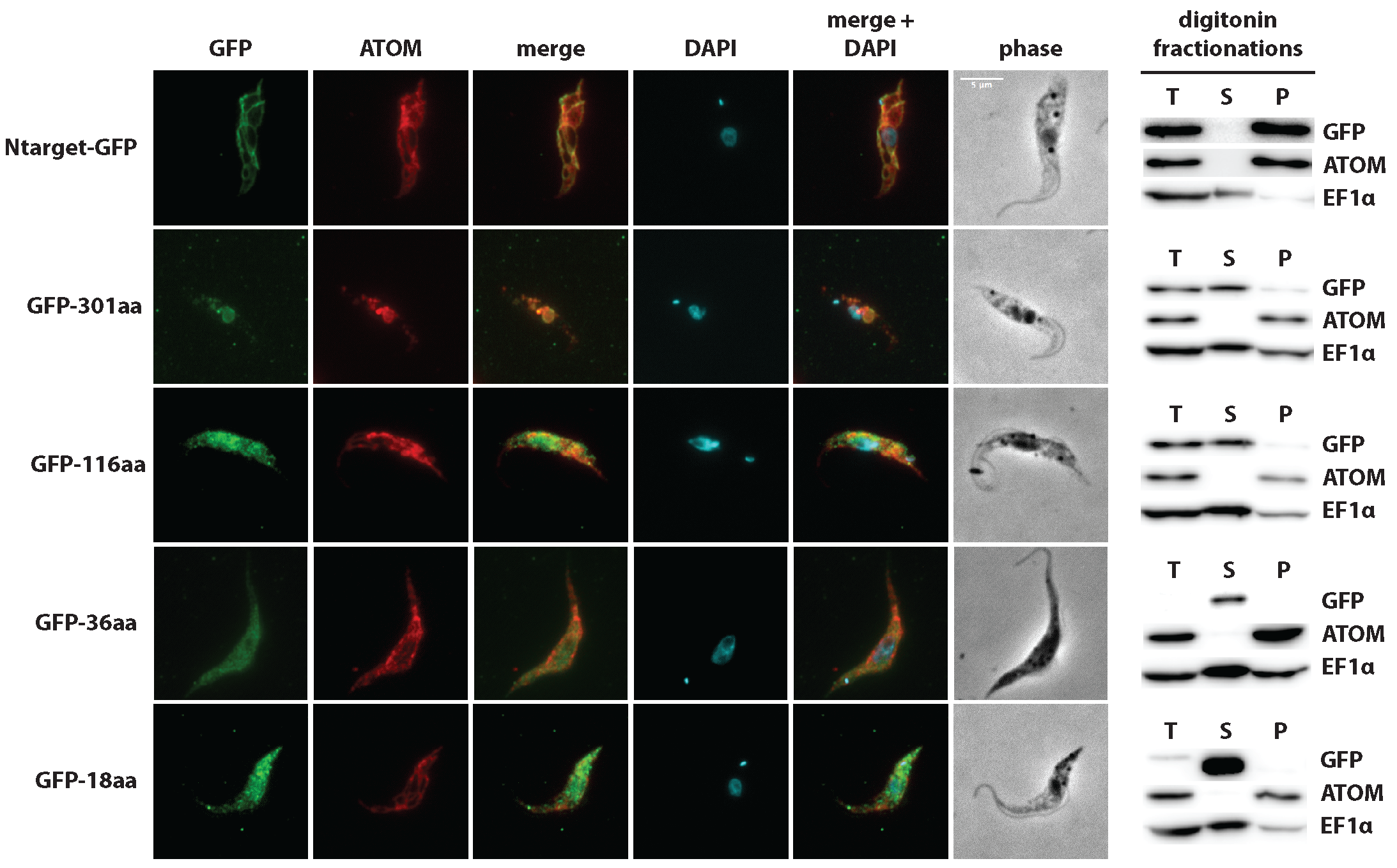

Supplement: S8 Fig — Expression was induced overnight. Immunofluorescence images show the localization of the GFP chimeras (visualized by anti-GFP antibody, green). ATOM is a mitochondrial marker protein, visualized by anti-ATOM antibody (red). DNA is stained with DAPI (cyan). On the right side of the immunofluorescence images, western blots of digitonin fractionations for each cell line are shown. ATOM and EF1α are used as fractionation controls. T, total cell lysate; S, supernatant; P, pellet. Ntarget-GFP: a control PCF cell line expressing GFP with an N-terminal mitochondrial targeting sequence of the Rieske iron-sulfur protein (Tb927.9.14160, 1−72 bp). This chimera co-localizes with ATOM and mitochondrial morphology is intact. GFP-301aa, GFP-116aa, GFP-36aa, GFP-18aa: PCF cell lines expressing GFP with the respective number of C-terminal amino acids of TAC102 fused to its C-terminus. GFP-301aa appears to co-localize with ATOM, but mitochondrial morphology is compromised (compare the localization of ATOM with the one seen in the Ntarget-GFP cell). GFP-116aa, GFP-36aa, GFP-18aa chimeras localize to the cytoplasm, but mitochondrial morphology as seen by staining for ATOM remains intact. (TIFF) [file ppat.1005586.s008.tiff]

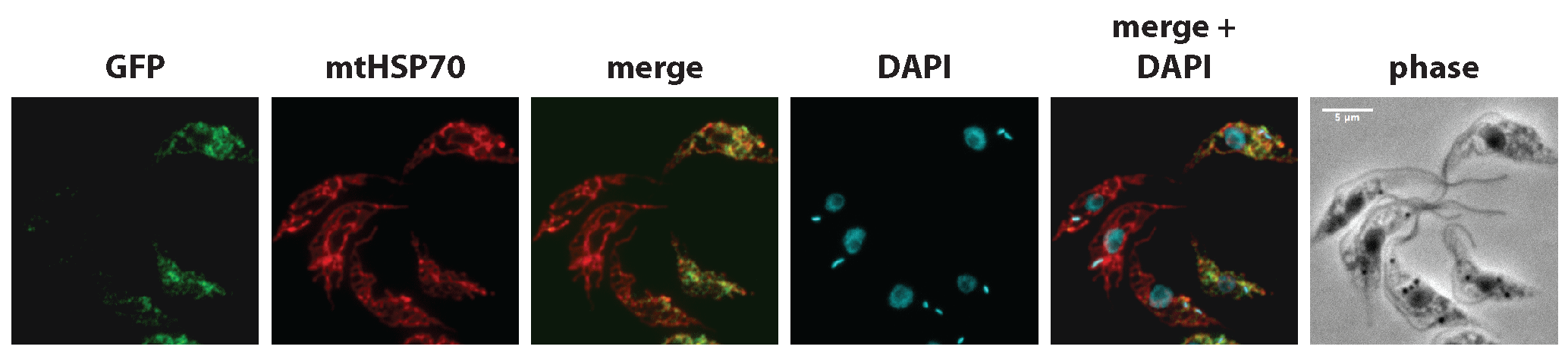

Supplement: S9 Fig — The GFP chimera is visualized by anti-GFP antibody (green). The mitochondrial heat-shock protein 70 (mtHSP70) is used as a mitochondrial marker (red). DNA is stained with DAPI (cyan). At this early time point of induction, few cells express GFP-301aa, which makes its detection by western blotting rather challenging. However, mitochondrial morphology as seen by mtHSP70 staining is unaffected, and GFP-301aa appears to co-localize with mtHSP70 in the cells that express GFP-301aa. (TIFF) [file ppat.1005586.s009.tiff]
