# Supplementary material for: Correction: TAC102 Is a Novel Component of the Mitochondrial Genome Segregation Machinery in Trypanosomes
Source: PLoS Pathog. 2016 Jul 1;12(7):e1005750. doi: 10.1371/journal.ppat.1005750 (PMC4930209; doi:10.1371/journal.ppat.1005750)
Supplement: S1 File — (PDF) [file ppat.1005750.s001.PDF]

PLOS | PATHOGENS

RESEARCH ARTICLE

TAC102 Is a Novel Component of the Mitochondrial Genome Segregation Machinery in Trypanosomes

Roman Trikin[1,2☯], Nicholas Doiron[1☯], Anneliese Hoffmann[1,2], Beat Haenni[3], Martin Jakob[1], Achim Schnaufer[4], Bernd Schimanski[1], Benoît Zuber[3], Torsten Ochsenreiter[1]*

1 Institute of Cell Biology, University of Bern, Bern, Switzerland, **2** Graduate School for Cellular and Biomedical Sciences, University of Bern, Bern, Switzerland, **3** Institute of Anatomy, University of Bern, Bern, Switzerland, **4** Institute of Immunology and Infection Research, University of Edinburgh, Edinburgh, Scotland, United Kingdom

☯ These authors contributed equally to this work.
* torsten.ochsenreiter@izb.unibe.ch

CrossMark
click for updates

OPEN ACCESS

Citation: Trikin R, Doiron N, Hoffmann A, Haenni B, Jakob M, Schnaufer A, et al. (2016) TAC102 Is a Novel Component of the Mitochondrial Genome Segregation Machinery in Trypanosomes. PLoS Pathog 12(5): e1005586. doi:10.1371/journal.ppat.1005586

Editor: Kent L. Hill, University of California, Los Angeles, UNITED STATES

Received: November 19, 2015

Accepted: March 30, 2016

Published: May 11, 2016

Copyright: © 2016 Trikin et al. This is an open access article distributed under the terms of the Creative Commons Attribution License, which permits unrestricted use, distribution, and reproduction in any medium, provided the original author and source are credited.

Data Availability Statement: All relevant data are within the paper and its Supporting Information files.

Funding: Funding was provided by the Swiss National Science Foundation (SNF) through a project grant to TO (SNF 140779), a career development award to BZ (SNF 139098) and BS (SNF 131959) and the Canton of Bern, Switzerland. The funders had no role in study design, data collection and analysis, decision to publish, or preparation of the manuscript.

Abstract

Trypanosomes show an intriguing organization of their mitochondrial DNA into a catenated network, the kinetoplast DNA (kDNA). While more than 30 proteins involved in kDNA replication have been described, only few components of kDNA segregation machinery are currently known. Electron microscopy studies identified a high-order structure, the tripartite attachment complex (TAC), linking the basal body of the flagellum via the mitochondrial membranes to the kDNA. Here we describe TAC102, a novel core component of the TAC, which is essential for proper kDNA segregation during cell division. Loss of TAC102 leads to mitochondrial genome missegregation but has no impact on proper organelle biogenesis and segregation. The protein is present throughout the cell cycle and is assembled into the newly developing TAC only after the pro-basal body has matured indicating a hierarchy in the assembly process. Furthermore, we provide evidence that the TAC is replicated *de novo* rather than using a semi-conservative mechanism. Lastly, we demonstrate that TAC102 lacks an N-terminal mitochondrial targeting sequence and requires sequences in the C-terminal part of the protein for its proper localization.

Author Summary

Proper segregation of the mitochondrial genome during cell division is a prerequisite of healthy eukaryotic cells. However, the mechanism underlying the segregation process is only poorly understood. We use the single celled parasite *Trypanosoma brucei*, which, unlike most model organisms, harbors a single large mitochondrion with a single mitochondrial genome, also called kinetoplast DNA (kDNA), to study this question. In trypanosomes, kDNA replication and segregation are tightly integrated into the cell cycle and thus can be studied alongside cell cycle markers. Furthermore, previous studies using electron microscopy have characterized the tripartite attachment complex (TAC) as a

Competing Interests: The authors have declared that no competing interests exist.

structural element of the mitochondrial genome segregation machinery. Here, we characterize TAC102, a novel trypanosome protein localized to the TAC. The protein is essential for proper kDNA segregation and cell growth. We analyze the presence of this protein using super resolution microscopy and show that TAC102 is a mitochondrial protein localized between the kDNA and the basal body of the cell's flagellum. In addition, we characterize different parts of the protein and show that the C-terminus of TAC102 is important for its proper localization. The data and resources presented will allow a more detailed characterization of the dynamics and hierarchy of the TAC in the future and might open new avenues for drug discovery targeting this structure.

Introduction

Trypanosoma brucei cells harbor a single mitochondrial organelle with a single genome, the kinetoplast DNA (kDNA), which consists of two types of circular DNA molecules, the maxi- and minicircles [1,2]. Maxicircles (~23 kb) encode subunits of the respiratory chain, a ribosomal protein and ribosomal RNAs [1]. Most of the maxicircle-encoded transcripts require posttranscriptional modifications by RNA editing [3–6]. This process involves several, well characterized large enzyme complexes, the editosomes [7], and small guide RNAs (gRNAs), which are encoded by the minicircles (~1 kb). The kDNA is a network of physically linked mini- (~5000) and maxicircles (~25) that forms a highly condensed, disk-like structure at the posterior end of the mitochondrion close to the basal body of the flagellum [1]. Replication of the kDNA occurs during the G1 phase of the cell cycle when the cells are characterized through the presence of one kDNA and one nucleus (1k1n) [8,9]. Prior to nuclear replication (S phase), the kDNA is segregated (2k1n) and, finally, after mitosis (G2/M) the cells contain two kDNAs and two nuclei (2k2n) [8,9]. More than 30 proteins have been characterized that are involved in the replication and compaction of the kDNA, however little is known about its segregation [1,2]. Also in yeast, the major model system for mitochondrial biology, knowledge about the mitochondrial genome segregation machinery is scarce [10–12]. There is evidence that the mitochondrial nucleoids are anchored via the inner and outer membranes of the organelle to the actin cytoskeleton and a number of proteins including Mmm1 and Mdm10/12/31/32/34 have been implicated in this process [10,13–16]. However most of these proteins are also involved in other processes related to mitochondrial morphology or mitochondrial ER contact sites [17–19], thus drawing final conclusions about their direct impact on mitochondrial genome segregation remains difficult.

The tripartite attachment complex (TAC)

Elegant electron microscopy analysis revealed a structure that connects the basal body with the kDNA disk, the tripartite attachment complex (TAC) [20]. The TAC consists of (i) the exclusion zone filaments, a region between the basal body and the outer mitochondrial membrane devoid of ribosomes; (ii) the differentiated mitochondrial membranes, which are inert to detergent extraction; and (iii) the unilateral filaments that connect the inner mitochondrial membrane with the kDNA spanning a region that has been described as the kinetoflagellar zone (KFZ) [1,2]. Although the basal body does not directly belong to the TAC structure, it is a key organizer in the *T. brucei* cell and the posterior anchoring point of the TAC [1,2,21]. A few markers for the basal body and the TAC have been described. Basal body markers include YL1/2 that recognizes the aggregation of non-polymerized tyrosinated tubulin in the transitional fibers of the mature flagellum [22], and BBA4 that recognizes an unknown protein in the pro- and mature basal bodies [23]. Furthermore, two components of the exclusion zone filaments

have been described. The monoclonal antibody MAB22 recognizes a cytoskeletal component of the exclusion zone filaments ranging from the proximal end of the basal body to the outer mitochondrial membrane [24]. The unidentified structure recognized by MAB22 seems to be insensitive to extraction by high concentrations of non-ionic detergents, which is consistent with the earlier descriptions of the TAC. The other known component of the exclusion zone filaments is a ~197 kDa protein (p197), which was shown to localize in the same region as MAB22 by immunofluorescence microscopy [25]. Depletion of p197 leads to a kDNA segregation phenotype where most cells are devoid of kDNA and a small number of cells accumulate a huge amount of kDNA [25]. For the differentiated membranes, a recently described rbarrel protein (TAC40) of the outer mitochondrial membrane (OM) has been demonstrated to be a TAC component [26]. Depletion of TAC40 leads to a phenotype similar to that described for p197. Additionally, electron microscopy studies demonstrated that the overall ultrastructure of the kDNA remains intact but daughter networks are not separated in cells depleted of TAC40 [26]. Another protein of the differentiated membrane is p166, historically the first TAC component to be described, which localizes to the inner mitochondrial membrane and its depletion leads to a phenotype similar to that described above for p197 and TAC40 [27]. Additionally, it was shown that the kDNA loss phenotype upon p166 RNAi is indeed a consequence of asymmetrical segregation rather than improper replication of the mitochondrial genome [27]. Potential candidates for anchoring the kDNA to the intermediate filaments are the two universal minicircle sequence binding proteins (UMSBP1 and UMSBP2) [28,29]. A homologue of these proteins in *Crithidia fasciculata* has been shown to specifically bind to two conserved sequences in the minicircles [30]. While the main function of the UMSBPs seems to be the initiation of minicircle replication, they might have additional functions in kDNA segregation. Currently it is unclear, however, if the kDNA segregation phenotype that is seen upon the loss of both UMSBPs is a consequence of the loss of minicircle replication or the proteins are directly involved in segregating the kDNA in trypanosomes. Aside from the UMSBPs, there are currently no other candidates for intermediate filament proteins.

Auxiliary factors of the TAC

AEP-1 is a mitochondrially encoded protein produced from the alternatively edited cytochrome c oxidase III mRNA. Overexpression of a recoded nuclear version of the C-terminally truncated AEP-1 (ΔC-AEP-1) led to a kDNA loss phenotype reminiscent of the phenotypes described above. Very likely this protein localizes to the inner mitochondrial membrane with a clear enrichment in the KFZ [31,32]. Recently, the thketoglutarate dehydrogenase E2 (αhKDE2) subunit, which is an essential Krebs cycle enzyme in the insect form trypanosomes, has been shown to be also important for proper kDNA segregation [33]. α3KDE2 seems to be localized throughout the mitochondrion and at the inner mitochondrial membrane and loss of this enzyme leads to a growth defect and a kDNA segregation phenotype.

In this study, we report a novel mitochondrial TAC protein (TAC102), which is likely part of the unilateral filaments. We show its localization throughout the cell cycle and characterize the phenotype which occurs during RNAi-induced loss of TAC102 and suggest a mechanism for the replication of the TAC structure. Additionally, we demonstrate that TAC102 has sequences in its C-terminus that are required for proper localization to the mitochondrial organelle and the TAC.

Results

TAC102 (Q57XN5; Tb927.7.2390) was discovered in an RNAi screen of proteins potentially involved in kDNA maintenance. To select candidates for the screen, we combined mRNA

expression data from the *T. brucei* cell cycle (S1D Fig) as well as proteomics data from the life cycle differentiation [34] and the MitoCarta [35]. TAC102 (i) is a predicted mitochondrial protein, (ii) is most highly expressed at the mRNA level in the G1 phase of the cell cycle when the new TAC is assembled, and (iii) its expression is decreased at the protein level during the differentiation to the non-replicative short stumpy cells. TAC102 is a single-copy gene encoding a 102.86 kDa (951aa) basic protein with pI 9.42 (S1 Fig). Orthologs of TAC102 can be found throughout the Kinetoplastea including *T. brucei*, *T. cruzi*, *Leishmania* spp., *Crithidia* sp. and *Phytomonas* sp. The C-terminal 120 amino acids of the protein are highly conserved among these species, but the middle part is variable and contributes to the varying pIs of the proteins. While the *Leishmania* spp., *Crithidia* sp. and *Phytomonas* sp. orthologs of TAC102 are acidic (pI around 4.5), the pI of the trypanosome orthologs that contain a lysine rich region (aa 653–756; 36% lysine) is above 9.4, suggesting different biochemical properties of these proteins. Previous studies detected posttranslational modifications of TAC102 –phosphorylation at positions 609 (T) and 614 (S) [36]. These residues are partially conserved among trypanosomes but not throughout the Kinetoplastea, however, little phosphoproteomics data is available for species other than *T. brucei* and *Leishmania* spp. (S1 Fig).

RNAi targeting TAC102 in bloodstream form (BSF) cells

Using RNAi which targets the ORF of TAC102, we depleted the transcript in bloodstream form (BSF) parasites and detected slower cell growth after three days of RNAi induction (Fig 1A). Seven days post RNAi induction the cells stopped growing entirely. The effectiveness of RNAi was shown by probing for the TAC102 mRNA and protein on northern and western blots, respectively (Fig 1A). Analysis of the DNA content of BSF cells by DAPI staining and fluorescence microscopy showed that approximately 75% of the cells had lost the kDNA two days after induction of RNAi (Fig 1B and 1C), while a small number of cells contained very large or “tiny” kDNAs (Fig 1B and 1D). The median intensity of the remaining kDNAs in the population increased by more than two fold 48 hours post RNAi induction based on DAPI fluorescence intensity (Fig 1D). Overall the amount of kDNA in the population decreased to ~45% of the wild type situation after two days of RNAi as measured by probing for the kDNA minicircles on Southern blots (Fig 1E). Also the number of cells properly segregating their kDNA (2k1n cells) dropped from ~15% in the uninduced population to less than 2% after two days of RNAi induction. Together with the appearance of very large kDNAs this suggests that loss of TAC102 has an impact on mitochondrial genome segregation rather than replication. In order to test if the loss of TAC102 also had influence on mitochondrial morphology, we stained the cells with antibody against the mitochondrial heat shock protein 70 (mtHSP70). During the first two days of RNAi induction we did not detect any changes in mitochondrial morphology (Fig 1B), except in cells that accumulated very large kDNAs; here an increase in organelle volume at the site of the kDNA was observed. Also the segregation of the mitochondrial organelle during cell division even in the absence of kDNA seemed to be unaffected (Fig 1B).

Essentially the same RNAi phenotype was observed in the insect form parasites (procyclic form, PCF; S2 Fig). Here we also carefully characterized the cells (<1%) that showed unequal segregation of the kDNA (S2I Fig). In the majority of these cells the enlarged kDNA was associated with the old basal body and flagellum, and it was positioned in most cases between the two nuclei. Thus, in both life cycle forms of *T. brucei* loss of TAC102 leads to kDNA missegregation rather than kDNA replication defect, which in turn leads to the loss of the mitochondrial genome in most cells. Based on our observations, we assume that in most cases the remaining kDNA is associated with the old basal body.

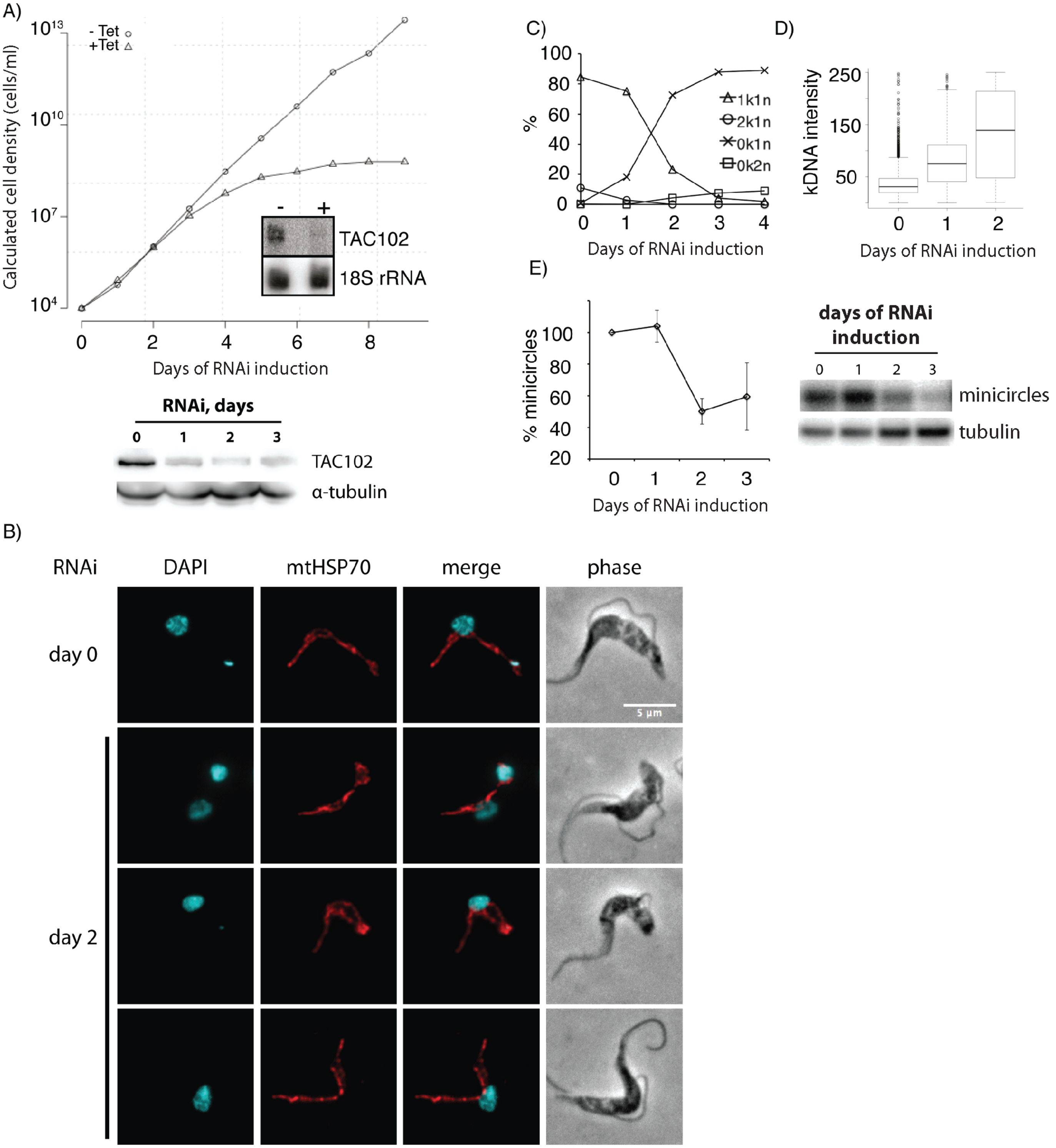

Fig 1. RNAi against TAC102 in BSF cells causes missegregation and loss of kDNA. A–growth curve of cells uninduced (–Tet) and induced (+Tet) for TAC102 RNAi. Inset: northern blot probed for TAC102 and 18S rRNA (loading control). RNA was isolated from cells that were uninduced (–) or induced for two days (+). The western blot under the growth curve demonstrates downregulation of TAC102 protein upon induction of RNAi. α Tubulin was used as a loading control. **B**–immunofluorescence images showing missegregation and loss of kDNA as well as the unchanged mitochondrial morphology upon induction of RNAi against TAC102. DNA is stained with DAPI (cyan). Mitochondria are visualized by staining for the mitochondrial heat-shock protein 70 (mtHSP70, red). Scale bar 5 he **C**–percentage of cells with different k-n-combinations within the course of TAC102 RNAi. **D**–intensity of the kDNA signal measured by DAPI staining. **E**–the relative amount of minicircle DNA decreases within the course of

TAC102 RNAi, according to Southern blotting. Tubulin was used for normalization and the amount of minicircle DNA in non-induced cells (day 0) was taken as 100%. A representative Southern blot is shown on the right side of the graph.

doi:10.1371/journal.ppat.1005586.g001

kDNA ultrastructure

In order to investigate the ultrastructure of the enlarged kDNA networks in BSF cells we employed transmission electron microscopy. The kDNA is organized in a disk-shaped structure which is situated close to the inner mitochondrial membrane. When the kDNA disk is viewed from the "side", the basal body can often be seen juxtaposed on the other side of the mitochondrial membranes (Fig 2A). Just prior to kDNA segregation, the kDNA assumes a kinked conformation (Fig 2B), which can be explained through the connection of the kDNA to the basal bodies and the movement of the new basal body around the old one. In BSF cells with enlarged kDNA networks two days after the induction of RNAi against TAC102 the kDNA generally maintains the striated ultrastructure of the disk (Fig 2C and 2D), however it does not assume a clear kinked structure (Fig 2C) and often the kDNA seems folded upon itself with additional smaller kDNA disks (Fig 2D). The median diameter of the sum of the striated kDNA disks from the enlarged networks was around 680 nm compared to 440 nm in the parental cell line (Fig 2G). In some cells, instead of a properly structured kDNA, we detected small patches of electron dense material (edm) lacking the typical striated appearance with a median diameter of 320 nm (Fig 2E). In the case of complete loss of the kDNA, the mitochondrial membranes remain in close proximity to the basal bodies and appear intact (Fig 2F). It also seems that the exclusion zone is unaffected since few or no ribosomes are present in this area (Fig 2F).

TAC102 is a component of the mitochondrial segregation machinery

Loss of kDNA is a frequent phenotype observed in trypanosomes when mitochondrial functions are affected. To test if loss of TAC102 has a direct or indirect effect on kDNA segregation, we made use of a recently described *T. brucei* BSF cell line that contains a single point mutation in the γ-subunit of the ATP-synthase (γL262P) and is able to shed its kDNA without any detectable growth defect [37]. RNAi targeting the ORF of TAC102 mRNA in this cell line led to the same phenotype as described above, i.e. loss of kDNA in the majority of cells and large/tiny kDNA networks in few cells; however, in this case the cells lacking kDNA continued to grow at wild type rates eventually leading to an akinetoplastic population without any defects in basal body or flagellar biogenesis. These experiments demonstrate that TAC102 is only essential in cells that require kDNA for proper growth (Fig 3A, 3B and 3C).

Localization of TAC102

In order to localize TAC102 in cells, the protein was tagged *in situ* at the N-terminus using a dual affinity tag PTP (*P*rotC-*T*EV-*P*rotA; [38]). Super-resolution confocal microscopy using a *ST*imulated *E*mission *D*epletion (STED) instrument showed co-localization of the N-terminally tagged TAC102 with MitoTracker in BSF cells (Fig 4A). Immunofluorescence microscopy detected the protein in the posterior part of the mitochondrial organelle between the basal body of the flagellum and the kDNA disk, in both BSF (Fig 4B) and PCF (Fig 4D) cells. The localization pattern of the tagged TAC102 was also confirmed in BSF and PCF cells by immunostaining with polyclonal and monoclonal antibodies against TAC102 (S2M and S2N Fig). Furthermore, biochemical fractionation using digitonin and differential centrifugation steps supported the mitochondrial localization of TAC102 (Fig 4C). We attempted to investigate the localization in more detail using different concentrations of digitonin to extract cells (Fig 4E).

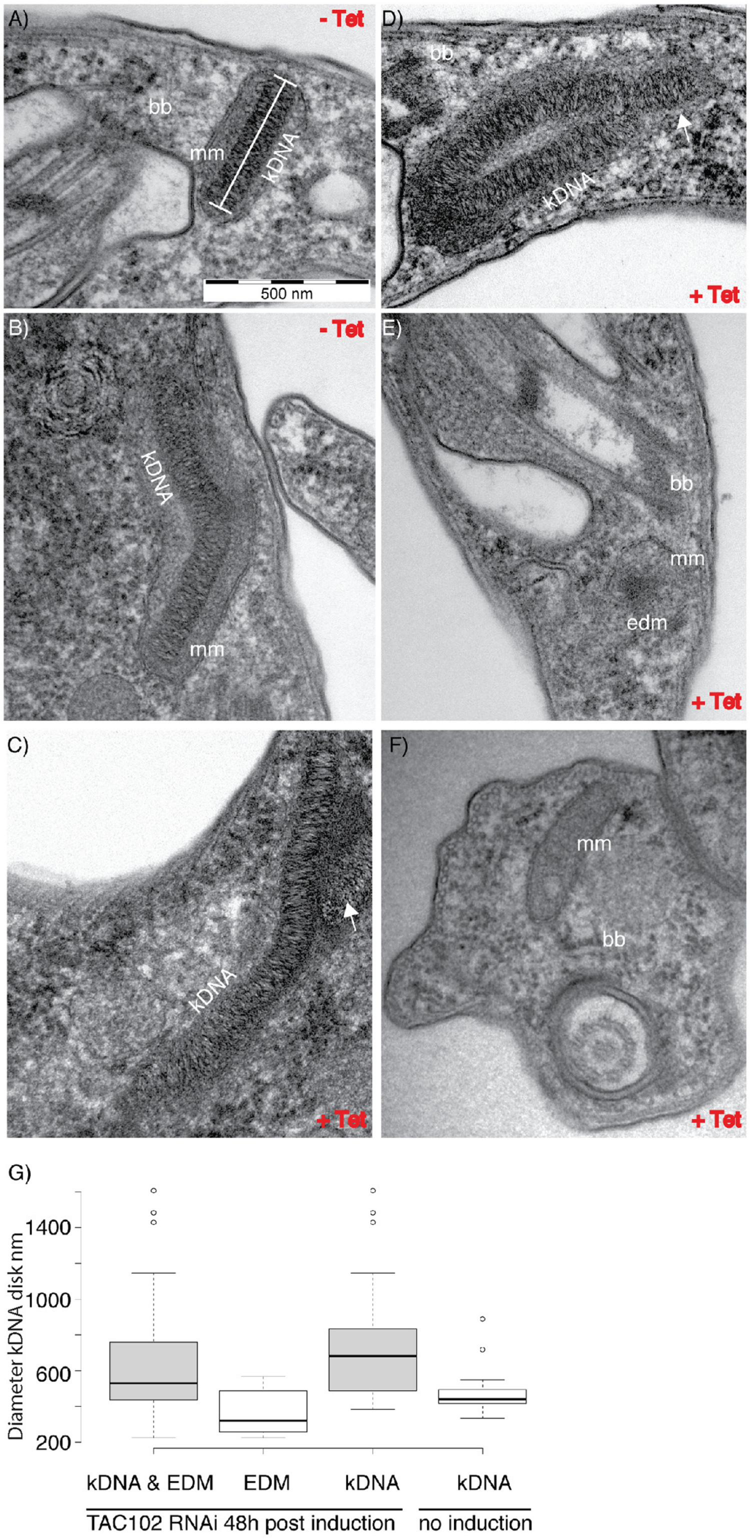

Fig 2. Ultrastructure of kDNA upon RNAi against TAC102 in BSF cells revealed by transmission electron microscopy. bb, basal body; mm, mitochondrial membrane; edm, electron dense material; –Tet, non-induced cells; +Tet, cells with TAC102 RNAi induced for two days. **A,B**–kDNA in a non-induced cells. **C, D**–examples of enlarged kDNA in cells induced for TAC102 RNAi for two days. The arrows point at additional patches of kDNA. **E**–example of a cell induced for TAC102 RNAi for two days that has lost the kDNA, instead

only a small patch of electron dense material (edm) can be seen surrounded by the mitochondrial membrane (mm) and localized in the proximity to the basal body (bb). **F**–an example of a cell induced for TAC102 RNAi for two days that has lost the kDNA and does not have any detectable electron dense material (edm); however, the mitochondrial membrane (mm) and the basal body (bb) are seen well. **G**–quantification of the kDNA diameter, measured as shown in **A** (white bar), in non-induced and induced cells.

doi:10.1371/journal.ppat.1005586.g002

TAC102 is observed in the soluble fraction at 0.1% detergent, resembling the behavior of lipid dehydrogenase (LipDH), a mitochondrial matrix protein. However, the archaic translocase of the outer mitochondrial membrane (ATOM) and the inner mitochondrial membrane protein cytochrome oxidase subunit 4 (COXIV), known to strongly associate with membranes, are solubilized only at 0.3% digitonin. From this we conclude that TAC102 is likely to localize in the mitochondrial matrix (Fig 4E). In order to test if TAC102 is indeed part of the TAC structure, we analyzed flagella isolated from BSF cells for the presence of the basal body, TAC102 and the kDNA using immunofluorescence microscopy (Fig 5). Under these conditions, >90% of the flagella showed a signal for the basal body and about 50% had kDNA attached to their posterior end as demonstrated by DAPI staining. Of the kDNA-positive flagella, >90% had a signal for TAC102 in close proximity to the basal body and the kDNA, indicating that TAC102, similarly to the previously described TAC40 and p166, is a component of the TAC. We also analyzed flagella isolated from PCF cells using immunofluorescence microscopy and western blotting (S3 Fig). Similarly to BSF cells, we observed that most flagella were positive for the presence of the TAC102 signal and the kDNA. In order to resolve the flagellar extract by SDS-PAGE and probe for TAC102 by western blotting, we had to treat the extracted flagella with DNAse I. TAC102 was found in both the flagellar and the soluble fractions, indicating that some part of the protein is solubilized by Triton X-100 used for flagella isolation, whereas a significant portion is retained at the flagella.

TAC102 during the cell cycle

A more detailed analysis of the localization of TAC102 using immunofluorescence microscopy shows that the protein is present throughout the cell cycle in whole cells (Fig 6). In the G1 phase, prior to kDNA replication, the TAC102 signal occupies a region between the basal body and the kDNA that is smaller than the kDNA structure as seen by DAPI staining (Fig 6A). Furthermore, based on staining of TAC102 and the mature basal body (YL1/2 antibody), the new TAC102 signal only appears after the new basal body matures (Fig 6B and 6C). During the nuclear S phase, the new basal body moves to its posterior position and TAC102 is present at both the old and the new basal body (Fig 6E and 6F). In G2/M, after kDNA segregation, TAC102 remains between the kDNA and the basal body as described for the situation in G1 (Fig 6G).

TAC replication

In order to test if the TAC is replicated *de novo* or by a semi-conservative mechanism, we induced RNAi against TAC102 in BSF cells for a short period (18 hours) and stained the cells with antibodies against TAC102 and the basal body (YL1/2) (S4 Fig). As expected, in non-induced cells each basal body is associated with a TAC102 signal and a kDNA. However, when TAC102 was depleted by RNAi for 18 hours, we observed cells that had two basal bodies, but just one TAC102 signal and one kDNA associated with it. The percentage of such cells in the population is 9.4%. Of such cells, more than 90% have the TAC102 signal/kDNA at the more anterior, old basal body. These experiments suggest that TAC102 is assembled *de novo* into the TAC.

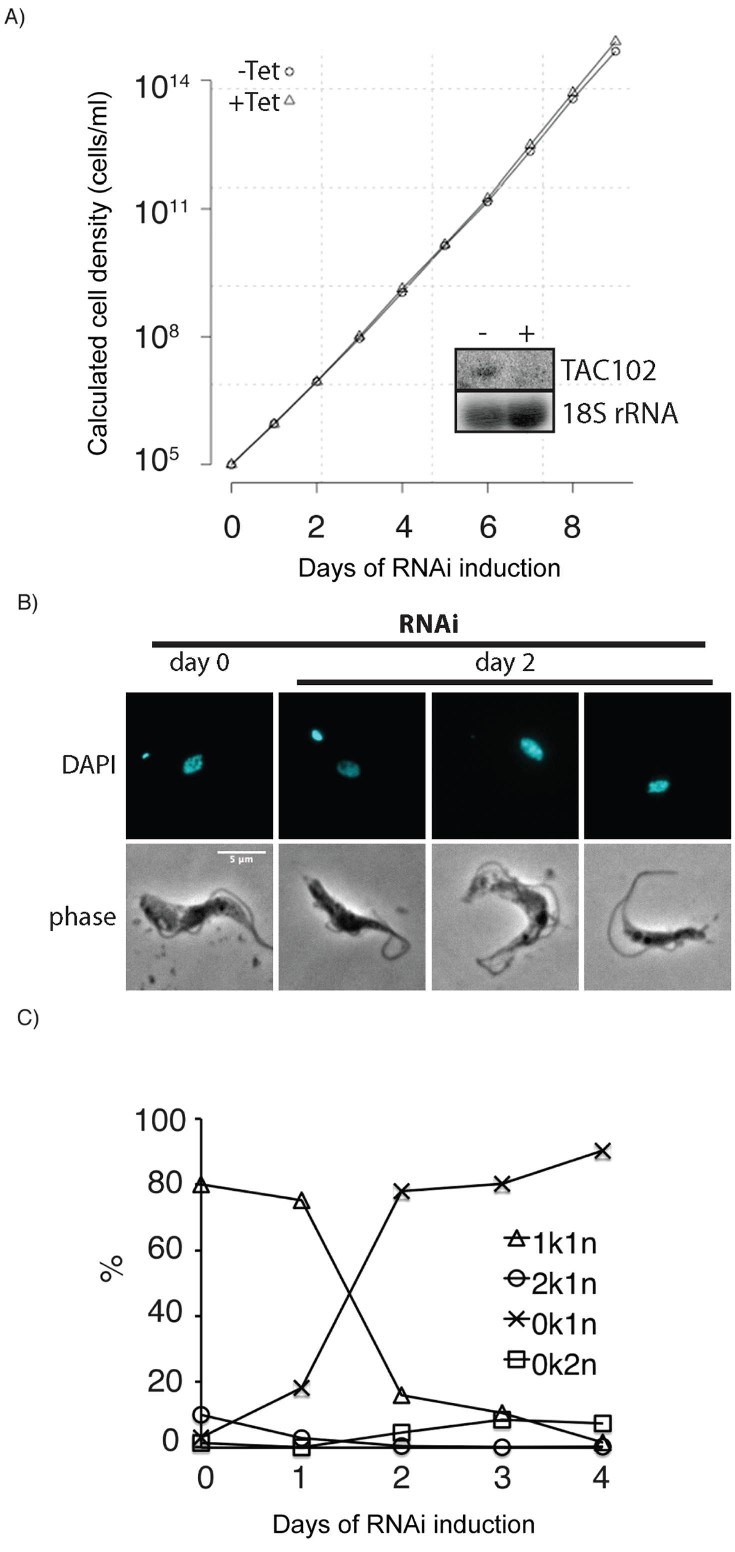

Fig 3. RNAi against TAC102 in γagainsBSF cells affects kDNA segregation but not cell growth. A–growth curve of cells uninduced (–Tet) and induced (+Tet) for TAC102 RNAi. Inset: northern blot probed for TAC102 and 18S rRNA (loading control). RNA was isolated from cells that were uninduced (–) or induced for two days (+). **B**–epifluorescence images (DAPI staining) of cells upon induction of RNAi against TAC102 for two days. Scale bar 5 n o **C**–percentage of cells with different k-n-combinations within the course of TAC102 RNAi.

doi:10.1371/journal.ppat.1005586.g003

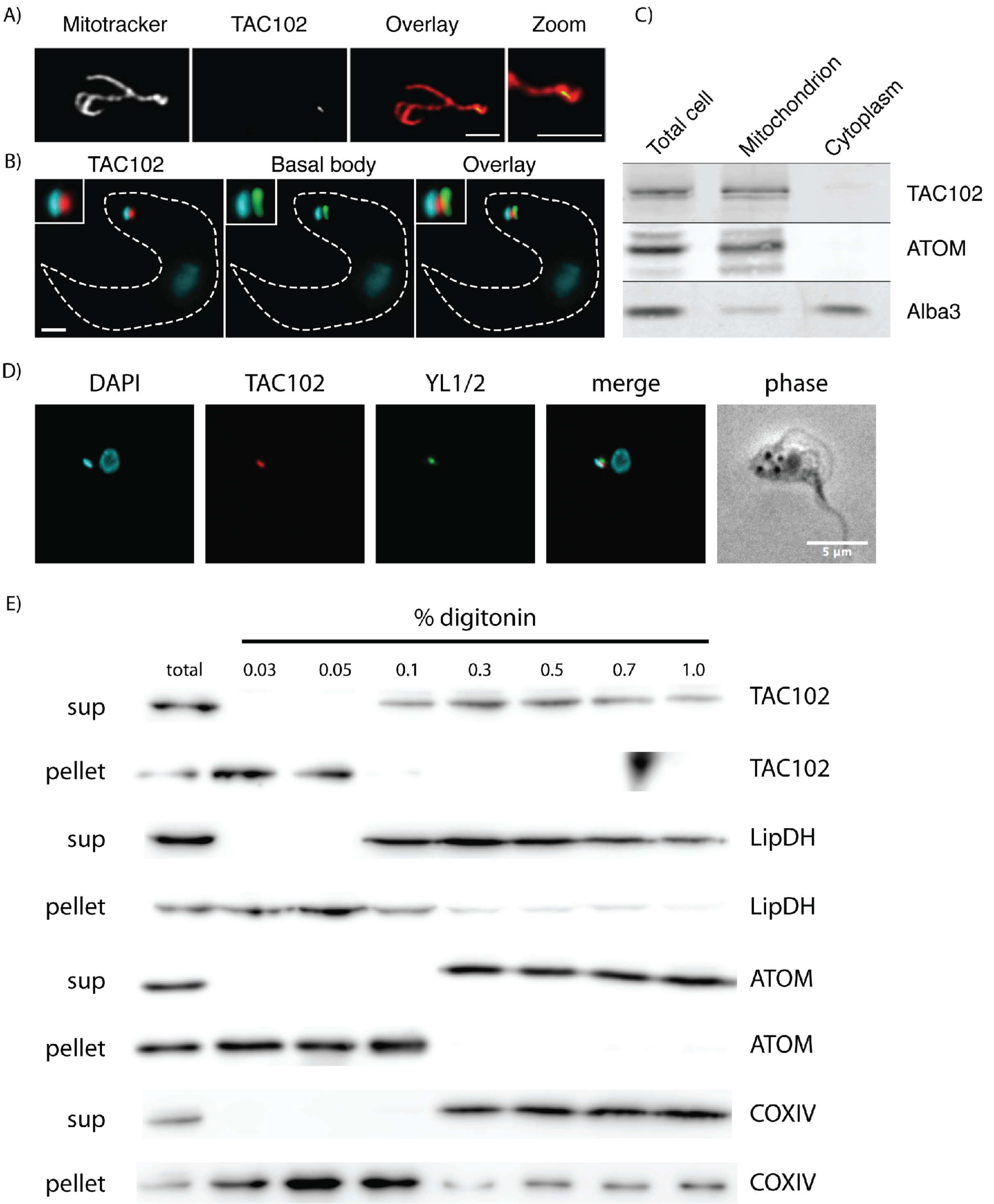

Fig 4. Localization of TAC102 in BSF and PCF *T. brucei*. A–STED microscopy image showing localization of N-PTP-TAC102 within the mitochondrion of a BSF cell. The mitochondria are stained with MitoTracker (gray, first image). The N-PTP-TAC102 is visualized by αTProteinA antibody (gray, second image). In the Overlay/Zoom, the mitochondrion is shown in red and TAC102 –in yellow. Scale bar 2 μm. **B**–immunofluorescence microscopy images showing the localization of N-PTP-TAC102 between the kDNA and the basal body of the flagellum in a BSF cell. DNA is stained with DAPI (cyan). The N-PTP-TAC102 is visualized by isProteinA antibody (red) and the basal body (YL1/2) is shown in green. The outline of the cell is shown with a white dashed line. Scale bar 2 μm, inset is a 200% zoom. **C**–western blot of a digitonin fractionation of BSF cells expressing N-PTP-TAC102. ATOM, a mitochondrial protein, and ALBA3, a cytosolic protein, are used as controls of the fractionation. **D**–immunofluorescence

microscopy images showing the localization of TAC102 between the kDNA and the basal body of the flagellum in a PCF cell. DNA is stained with DAPI (cyan). TAC102 is visualized by muTAC102 antibody (red) and the basal body is visualized by α-YL1/2 antibody (green). Scale bar 5 y (**E**–western blots of digitonin fractionations from PCF cells. Fractionations were performed with different concentrations of the detergent. total, total cell lysate; sup, supernatant. LipDH, lipid dehydrogenase, a mitochondrial matrix protein; ATOM, archaic translocase of the outer mitochondrial membrane; COXIV, cytochrome oxidase subunit 4, an inner mitochondrial membrane protein.

doi:10.1371/journal.ppat.1005586.g004

Characterization of TAC102 domains

Since TAC102 does not contain a detectable mitochondrial targeting signal at the N-terminus we aimed to characterize the region of the protein necessary for its proper localization to the TAC. For this we overexpressed inducible ectopic copies of N-terminally myc-tagged TAC102 in PCF parasites that were (i) truncated at the N-terminus (deletion of the first 200 aa, mycΔN-TAC102) or (ii) the C-terminus (deletion of aa 650–951, mycΔC-TAC102), as well as (iii) the full-length TAC102 (myc-TAC102) and followed their localization in the cell by fluorescence microscopy and biochemical digitonin fractionation. The truncations cover most of the conserved regions of TAC102 in the N- and C-terminus. Myc-TAC102 localizes to the position of the endogenous protein as determined by immunofluorescence microscopy and western blotting of digitonin fractionations (Fig 7 and S5 Fig). Furthermore, overexpression of the N-terminally tagged TAC102 does not lead to any detectable growth or cell cycle phenotype (Figs 7, S5 and S6A). The N-terminally truncated TAC102 (mycΔN-TAC102) localizes to the

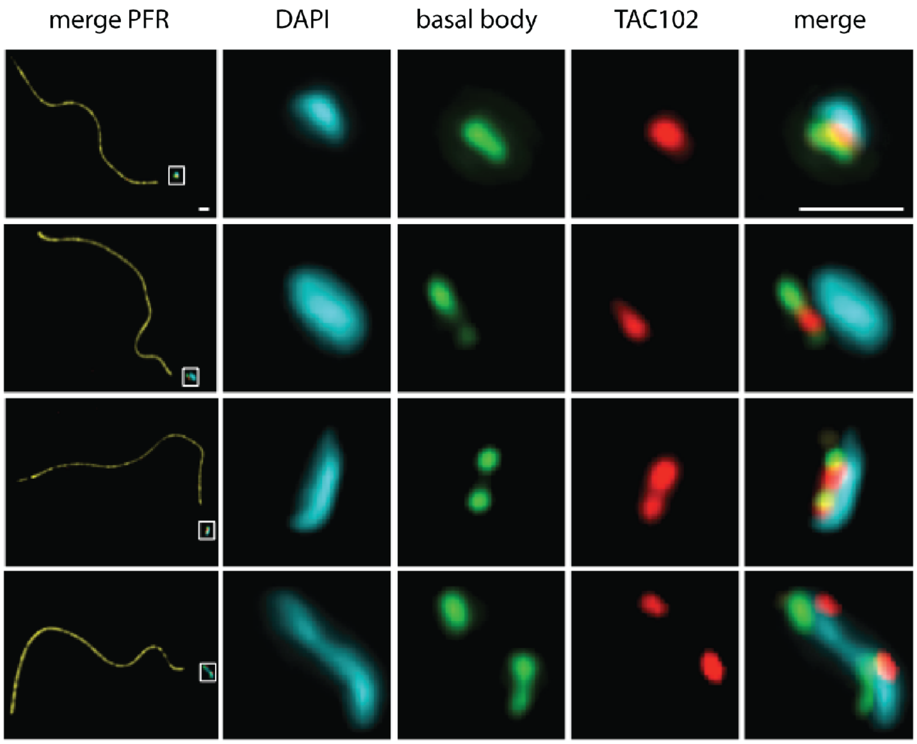

Fig 5. TAC102 remains associated with the flagellum after flagellar extraction of BSF cells. Immunofluorescence analysis was performed with flagella isolated from BSF cells that express N-PTP-TAC102. The flagella are stained with PFR antibody (yellow). The structure at the end of the flagellum (in a white square box) is enlarged to show the kDNA (stained with DAPI) in cyan, the basal body (visualized with α-BBA4 antibody)–in green and N-PTP-TAC102 (detected by α-ProteinA antibody)–in red. Scale bar 1 μm.

doi:10.1371/journal.ppat.1005586.g005

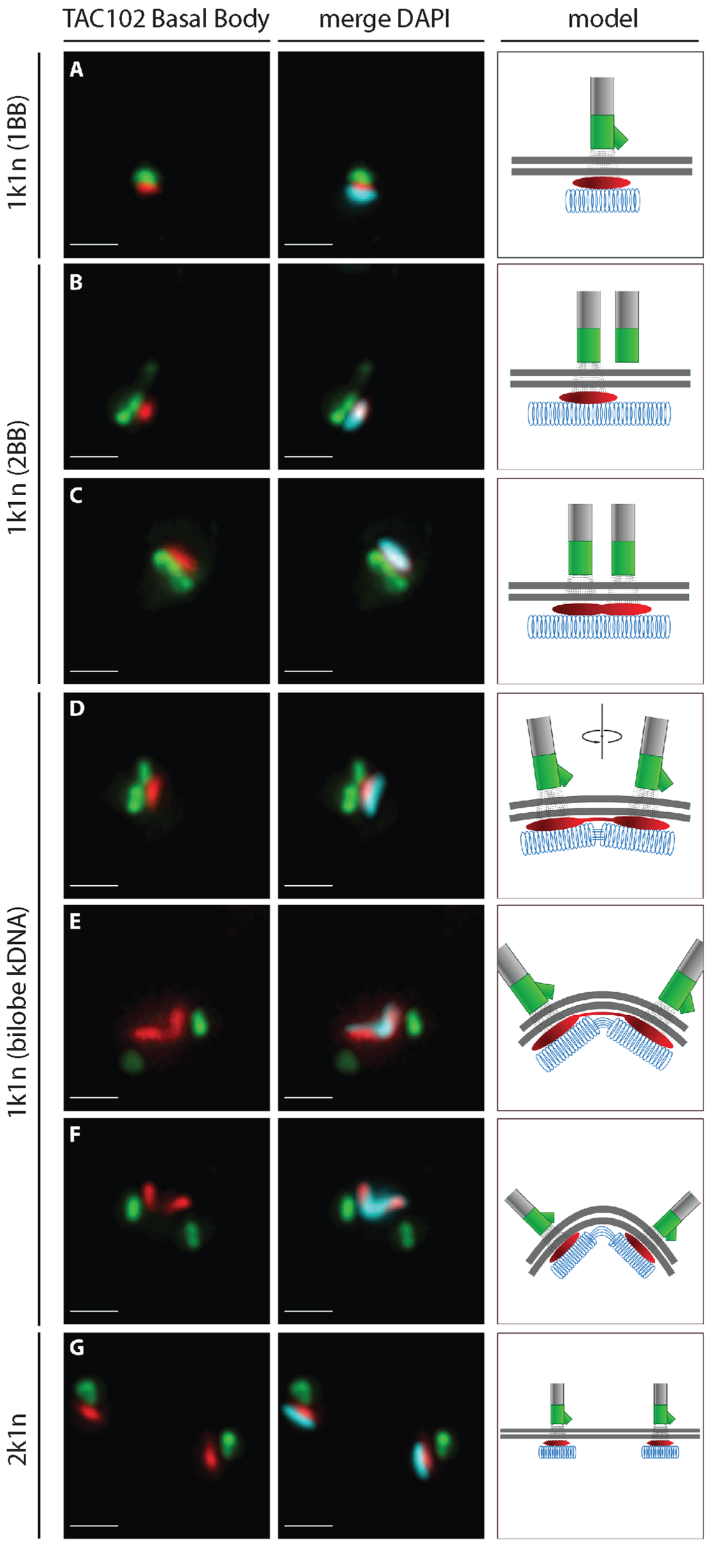

Fig 6. Localization of TAC102 in BSF *T. brucei* during different stages of kDNA replication. Immunofluorescence images show the localization of N-PTP-TAC102 (detected by α-ProteinA antibody, red), the basal body (YL1/2, green) and the kDNA (stained with DAPI, cyan). A schematic model corresponding to each situation is shown on the right side of the images. Panels **A** to **G** represent different stages during kDNA replication. Scale bar 1 ale

doi:10.1371/journal.ppat.1005586.g006

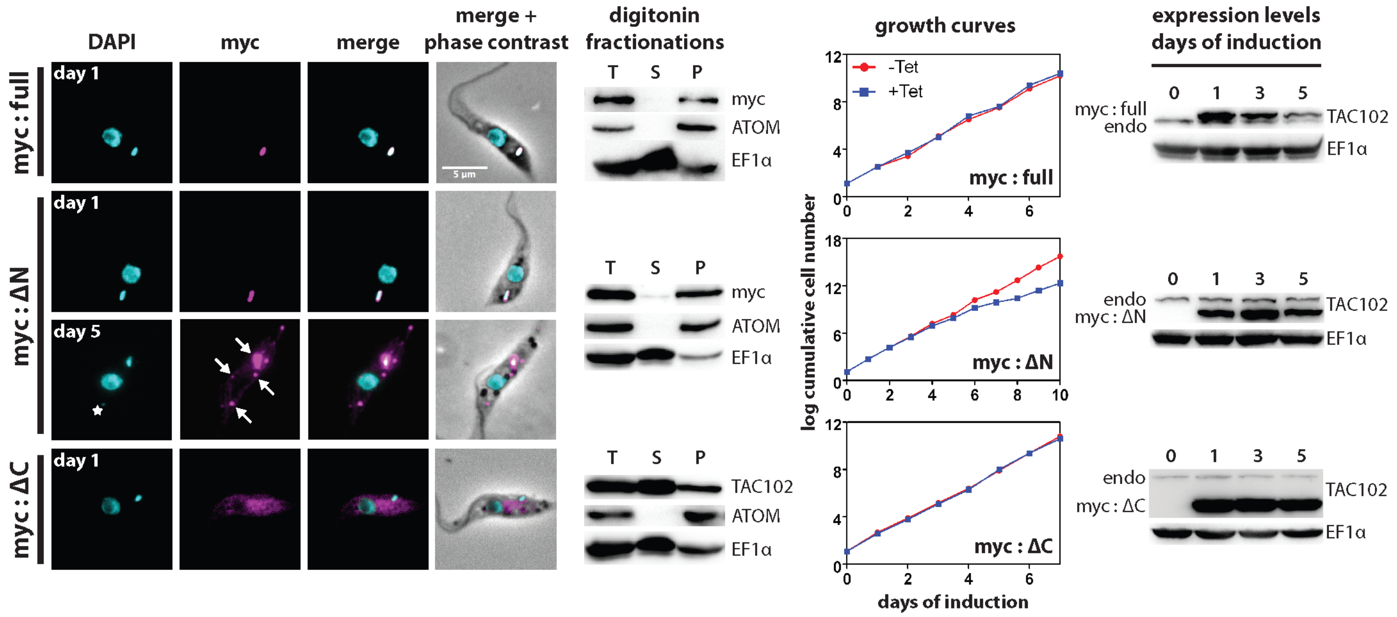

Fig 7. Analysis of truncated versions of TAC102 upon their overexpression. The full-length (myc:full), N-terminally (myc:, N or C-terminally (myc:r) truncated TAC102 was expressed with a triple myc-tag at the N-terminus in PCF cells. Immunofluorescence images show the localization of the tagged proteins. DNA is stained with DAPI (cyan) and myc-tagged proteins (visualized by α-myc antibody) are shown in magenta. The star (myc:my, day 5) indicates an ancillary kinetoplast and the arrows indicate accumulation of the protein. Scale bar 5 cateFor each of the three cell lines, western blots of digitonin fractionations are shown. ATOM (a mitochondrial protein) and EF1) (a cytosolic protein) are used as fractionation controls. T, total cell lysate; S, supernatant; P, pellet. Fractionations were performed on day 1 post induction. The growth curves show cell growth upon expression of the tagged proteins. The western blots on the right side of the growth curves show expression levels of the tagged versions of TAC102 in comparison to those of the endogenous protein (endo). EF1α is used as a loading control.

doi:10.1371/journal.ppat.1005586.g007

TAC, however after five days of overexpression 25% of the cells show additional foci of mycΔN-TAC102 that are in the mitochondrial organelle as confirmed by biochemical fractionation; some of the TAC102 foci co-localize with small ancillary kinetoplasts (Fig 7 and S5 Fig). Most of the cells with ancillary kDNAs, >80%, have one "extra" kDNA per cell (S6B Fig). After five days of induction we also could detect a reduction in the number of cells in G1 (1k1n) as well as an increase in cells without kDNA (10%; S6A Fig). Furthermore, mycΔN-TAC102 cells displayed a very weak growth defect that starts six days post induction of overexpression of the protein. Thus the N-terminus of TAC102 is not required for import into the mitochondrion and targeting to the TAC but prolonged overexpression of the N-terminally truncated version leads to additional TAC102 foci, some of which associate with ancillary kinetoplasts. The C-terminally truncated TAC102 (mycΔC-TAC102), on the other hand, does not localize to the mitochondrion or the TAC but accumulates in what is likely the cytoplasm (Fig 7 and S5 Fig), indicating that the C-terminus may be important for proper localization to the organelle. This was also supported by the biochemical digitonin fractionations that showed the majority of TAC102 in the supernatant fraction (Fig 7).

Since the mycΔN-TAC102 localizes to the TAC, we wanted to test if the mutant protein can complement the depletion of the endogenous TAC102. For this we used RNAi targeting the 3'-UTR of the endogenous TAC102 mRNA (S2D–S2I Fig). In this experiment the ectopically expressed mycΔN-TAC102 or myc-TAC102 mRNAs contained the aldolase 3'-UTR and thus were not affected by RNAi targeting the native TAC102 3'-UTR (Fig 8). Myc-TAC102 is able to partially rescue the TAC102 RNAi phenotype. The growth defect is delayed by two days, when compared to the cells without complementation (Fig 8 and S2D Fig). Most importantly,

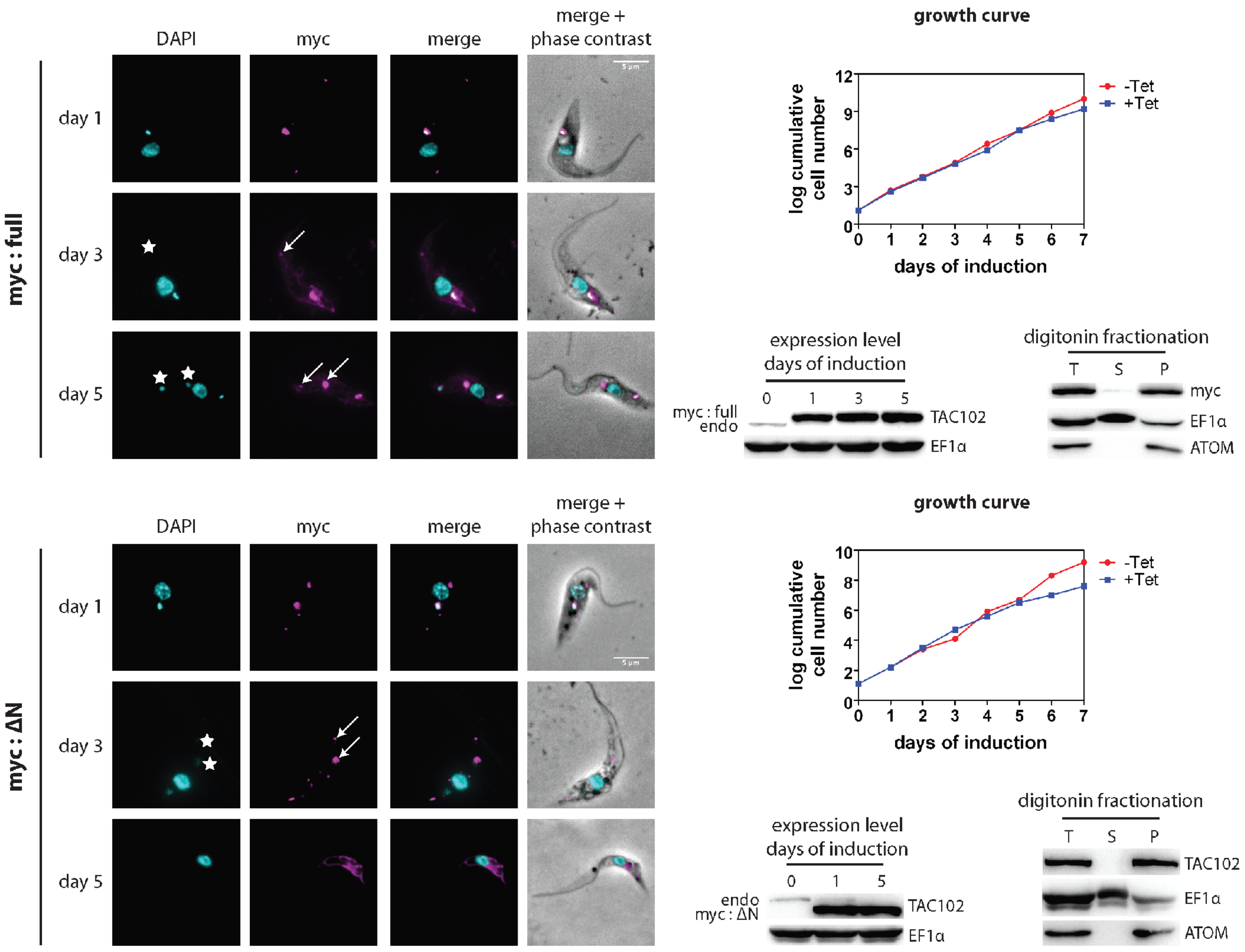

Fig 8. The myc:full version of TAC102 is able to partially compensate for the loss of the endogenous TAC102 but the myc:ΔN is not. The full-length (myc:full) or the N-terminally truncated (myc:yc: version of TAC102 was expressed with a triple myc-tag at the N-terminus in PCF cells that contain a construct for inducible RNAi against the 3'-UTR of the endogenous TAC102. Immunofluorescence images show the localization of the tagged proteins. DNA is stained with DAPI (cyan) and myc-tagged proteins (visualized by α-myc antibody) are shown in magenta. Arrows show additional foci of TAC102 accumulation. Stars show ancillary kDNAs. Scale bar 5 addiFor each of the two cell lines, growth curves of the cells upon expression of the tagged proteins/knockdown of the endogenous TAC102 are shown on the right side of the immunofluorescence images. Under the growth curves (left side) are western blots that show expression levels of the tagged versions of TAC102 in comparison to those of the endogenous protein (endo). EF1o)stern d as a loading control. Under the growth curves (right side) are western blots of digitonin fractionations. ATOM (a mitochondrial protein) and EF1α (a cytosolic protein) are used as fractionation controls. T, total cell lysate; S, supernatant; P, pellet. Fractionations were performed on day 5 of induction.

doi:10.1371/journal.ppat.1005586.g008

even after five days of RNAi induction and simultaneous overexpression the majority of cells (>98%) still contain kDNA, albeit in many cases additionally to the proper posterior location also at non-conventional positions within the mitochondrion (Fig 8 and S6 Fig). These ancillary kinetoplasts co-localize with additional TAC102 punctae (Fig 8). In this cell line, more than 60% of the cells with ancillary kinetoplasts had just one "extra" kDNA structure per cell after five days of induction (S6B Fig). The N-terminally truncated TAC102 (mycΔN-TAC102), on the other hand, is unable to rescue the kDNA loss phenotype induced by RNAi against the endogenous TAC102. On day five post induction >60% of cells have lost their kDNA, while 14% show ancillary kDNAs (Fig 8 and S6 Fig). In the cells that have "extra" kDNA, we observe

one, two or three ancillary kinetoplasts per cell appearing in the population with similar frequencies, and we also sometimes detect cells with even more "extra" kDNA structures (S6B Fig). In summary, we have shown that the N-terminus of TAC102 is not required for its proper localization to the mitochondrion and the TAC, however it is required for proper function since the N-terminal deletion mutant of TAC102 is unable to rescue the loss of the endogenous protein. Furthermore, even though the full length N-terminally tagged TAC102 partially rescues the TAC102 RNAi phenotype, it leads to extra TAC102 and kDNA foci, indicating that the proper function of the protein is compromised by the myc-tag.

The C-terminus of TAC102 is important for its localization to the mitochondrion

Since the C-terminal deletion mutant of TAC102 (mycΔC-TAC102) mislocalized to the cytoplasm, we hypothesized that the targeting signal for mitochondrial import of TAC102 is in the C-terminal 301 aa of the protein. This was supported by bioinformatics analysis that predicted the last 18 and 36 aa of TAC102 to form amphipathic helices (S7 Fig), a hallmark of N-terminal targeting sequences and one C-terminally targeted protein in yeast [39–41]. Furthermore, the last 116 aa of TAC102 are highly conserved among Kinetoplastea supporting the hypothesis that this region might be important for the function of the protein.

Thus in an attempt to investigate the potential role of the C-terminal part of TAC102 in mitochondrial import, we created four PCF cell lines expressing different parts of the C-terminus of TAC102 that were C-terminally fused to GFP. As a positive control, we used GFP with a known N-terminal targeting sequence. We induced and followed the expression of the chimeric proteins by fluorescence microscopy and western blotting after digitonin fractionation (S8 Fig). While the GFP containing the N-terminal mitochondrial targeting signal was imported into the mitochondrion, we found mostly cytosolic localization when we added 18, 36, or 116 of the TAC102 C-terminus to the GFP and induced expression of the protein overnight. However, when we added the last 301 aa of the C-terminus of TAC102 to GFP, we noticed (i) co-localization with the mitochondrial marker ATOM and (ii) that the mitochondrial network morphology was compromised. In order to further investigate this observation, we expressed this GFP construct for a shorter period of time (Fig 9). After 1 and 2 hours of induction, individual cells started to express the GFP chimera that co-localized with the mitochondrial marker but no change in mitochondrial morphology could be detected. However, as early as 3 and 4 hours post expression of the GFP chimera the organelle morphology started to change. We were unable to corroborate immunofluorescence microscopy data by digitonin fractionations, as only few cells expressed GFP-301aa at these early time points (S9 Fig) and the overall level of expression was below detection by western blotting. Thus overexpression of the 301 C-terminal amino acids from TAC102 fused with GFP eventually leads to changes in the morphology of the mitochondrial organelle, while the protein localizes, at least partially, to the organelle. From these experiments we conclude that the C-terminus is important for localization to the mitochondrial organelle but is unable to target the protein to the TAC.

Discussion

RNAi against TAC102 in BSF and PCF cells (Fig 1 and S2 Fig) leads to missegregation of the kDNA and eventually to kDNA loss in a large part of the population. While DAPI staining reveals a dramatic loss of kDNA already two days post induction of RNAi in BSF cells (>75% 0k1n cells), the detection of minicircles by Southern blotting shows a much less dramatic loss at this time. The apparent discrepancy can be explained by (i) the increase of kDNA in some cells that retain the kinetoplast (Fig 1D) and (ii) the appearance of small kDNA structures

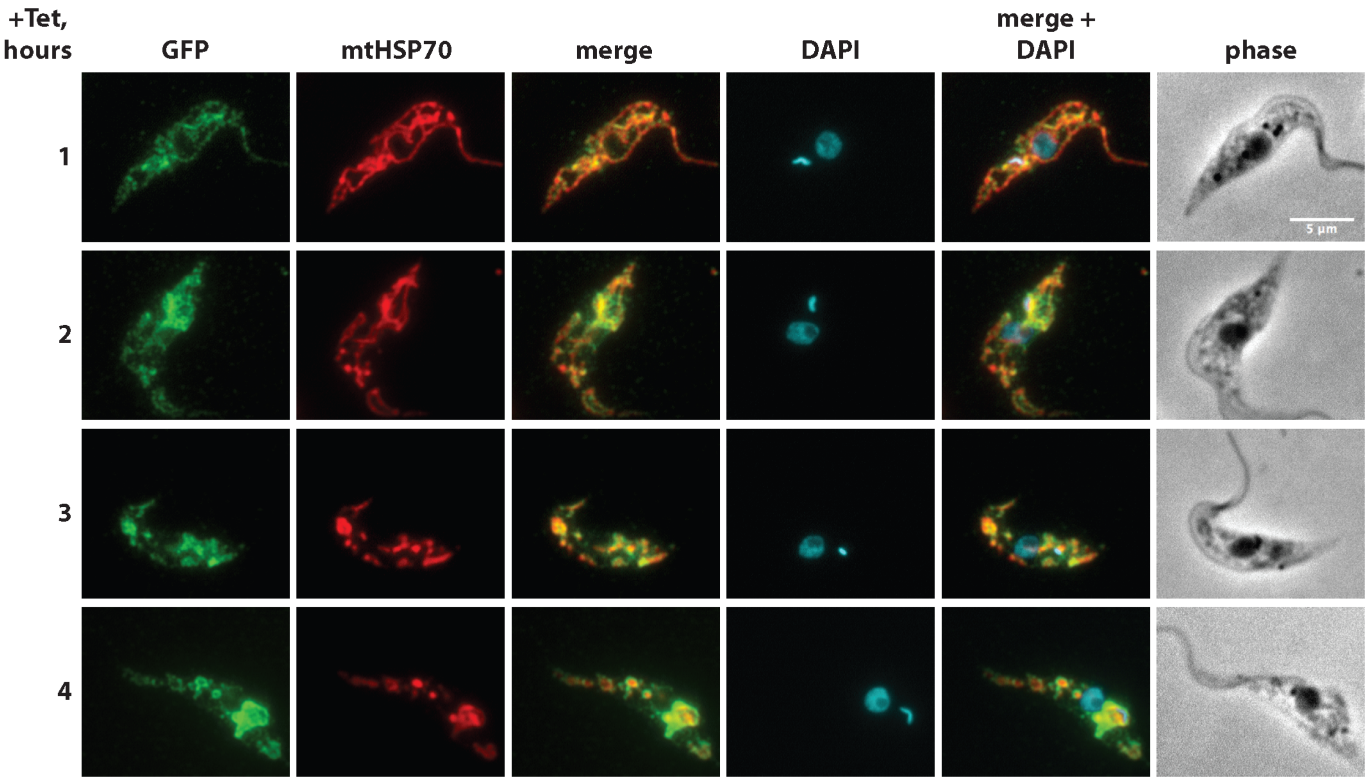

Fig 9. Ectopic expression of GFP-301aa fusion protein in PCF cells at early time points of induction. GFP-301aa is a chimeric protein where the last 301 aa of TAC102 are fused to the C-terminus of GFP. Expression of GFP-301aa was induced for 1, 2, 3 or 4 hours. Immunofluorescence images show the localization of GFP-301aa (visualized by α-GFP antibody, green). The mitochondrial heat-shock protein 70 (mtHSP70) is used as a mitochondrial marker (red). DNA is stained with DAPI (cyan). Scale bar 5 een

doi:10.1371/journal.ppat.1005586.g009

(Figs 1B and 2), which sometimes may be too small to be identified on microscopic images of DAPI stained cells but still can be detected by Southern blotting.

The effect of RNAi against TAC102 on kDNA segregation is further illustrated by our TEM experiments (Fig 2). Upon loss of TAC102, we observe enlarged kDNAs, retaining the striated structure and the overall kinetoplast morphology seen in wild type cells. Sometimes additional small but well structured kDNAs were attached to the non-segregated networks, similarly to what has been described for p166 and TAC40 [26,27]. Interestingly, we also observed small patches of electron dense material (edm) in some cells. We assume that these could be the “small” kinetoplasts, which we also detect in epifluorescence microscopy upon the knockdown of TAC102 (Fig 1B). The edm does not have the typical appearance and the structure of kDNA and is never seen together with regular, well-structured kDNAs in the same cell. We speculate that, when the kDNA size is reduced past a certain threshold after improper segregation, the network loses some structural proteins and is not able to condense properly. We have shown that TAC102, similarly to p166 and TAC40, is associated with isolated flagella from BSF and PCF cells, indicating that the protein is tightly bound to the TAC (Fig 5 and S3 Fig). However, in biochemical fractionations with increasing concentrations of digitonin the protein is readily soluble, similarly to a typical matrix protein (Fig 4E). So how can TAC102 remain at flagella upon treatment with a strong detergent such as Triton X-100, used for flagellar isolation, and be relatively soluble upon digitonin fractionation? We speculate that TAC102 forms a multimeric structure in the TAC which is partially soluble, probably due to a different degree of association with other components. Alternatively, the difference in the solubilization by

digitonin and Triton X-100 might also be explained by the difference in the chemical nature of the detergents.

Based on the RNAi studies, TAC102 is a component of the mitochondrial genome segregation machinery and loss of TAC102 does not alter mitochondrial morphology (Fig 1B) or the ability to properly segregate the organelle during cell division, similarly to what we recently described for TAC40 [26], an outer mitochondrial membrane component of the TAC. Thus, mitochondrial genome segregation and organelle segregation are two independent processes and failure to properly segregate the mitochondrial genome does not directly impact cell division. The apparentexclusive function of TAC102 in kDNA segregation is supported by our experiments in the γL262P mutant trypanosomes (Fig 3), a cell line that is able to compensate for mitochondrial genome loss through a mutation in the γ-subunit of the ATP synthase [37]. When depleted of TAC102, the γL262P cell line shows the kDNA loss phenotype as described above, however, without any growth defect. This demonstrates that in the γL262P TAC102 RNAi cells no essential function is compromised. The exclusive function of TAC102 and the recently described protein TAC40 [26] in kDNA segregation is surprising since in yeast, for example, all known segregation factors are also involved in other functions, including maintenance of ER-mitochondrial contact sites and organelle morphology [17–19]. This makes trypanosomes a very attractive model system to study mitochondrial DNA segregation, as components of the kDNA segregation machinery are unlikely to be implicated in other processes.

Interestingly, the phenotypes that are observed upon TAC102, p166, TAC40 or p197 depletion are very similar in kinetics and extent of kDNA loss [25–27]. Additionally, for TAC102 and TAC40 it has now been demonstrated that their function is exclusively associated with mitochondrial genome maintenance. Based on the similarities in loss of function phenotypes we speculate that the same is true for p197 and p166. Thus these four components of the TAC behave differently from the previously described TAC-associated components ACP, AEP-1 or ssociatthat are also required for proper kDNA maintenance but either have additional functions, like α-KDE2 [33], or are more indirectly involved in the segregation, like ACP that is crucial for proper lipid biogenesis [42]. AEP-1 is a special case since it is the only mitochondrially encoded component reported so far and, while it showed enriched localization at the TAC, it was also detected throughout the mitochondrial organelle, hinting at other potential functions of this protein [31,32]. Thus we consider TAC102, TAC40, p166 and p197 to be the "core" components of the TAC.

The TAC102 RNAi experiments show that enlarged kDNAs mostly associate with the old basal body and cells that retain two kinetoplasts (2k2n cells), although very rare, mostly show unequal segregation of the mitochondrial genomes (S2I Fig), a phenomenon that was previously also observed in cells depleted of p166 [27]. The specific loss of the kDNA—new basal body connection argues that the old basal body–kDNA connection is, at least initially, not affected by RNAi targeting TAC102 or p166. Thus the TAC that is associated with the new basal body is likely assembled as a *de novo* structure rather than replicated in a semi-conservative mode, in which case we would have expected a random loss of the basal body–kDNA connection. This is further corroborated by our experiments demonstrating that the loss of TAC102 upon RNAi preferentially occurs at the new basal body–kDNA connection, leaving the old basal body–kDNA connection intact (S4 Fig).

A peculiar observation was the small ancillary kDNAs that appeared upon expression of several tagged TAC102 constructs (myc:ΔN-TAC102; myc:ΔN-TAC102 and myc-tagged full length TAC102 both in the absence of the endogenous protein). These results suggest that the N-terminus of TAC102 is important for the connection to upstream components of the TAC (closer to the basal body) and its deletion or obstruction by a tag leads to a partial loss of

function of the protein, namely the proper localization at the TAC. Furthermore, the results indicate that the C-terminal part of the protein in the absence of the N-terminus is sufficient to either directly connect to kDNA or initiate the assembly of the downstream components that connect to the kDNA and thus initiate the appearance of the ancillary kinetoplasts that have lost the connection to the upstream components of the TAC and, consequently, the basal body. Ancillary kinetoplasts naturally occur in several Kinetoplastea species but the frequency of their appearance in *T. brucei* is very low and under normal culture conditions these “extra” kDNAs are rarely detected [43]. However, there are examples where depletion or overexpression of mitochondrial proteins leads to additional kDNA structures in the mitochondrion. In Tim17 RNAi cells up to 10% of the population accumulate extra kDNA [44], but since Tim17 is a protein import component, this effect is likely indirect and occurs due to the loss of kDNA segregation/replication factors, e.g. POLIB and POLIC, which, if depleted by RNAi, also lead to the appearance of ancillary kDNA structures [45]. The depletion of the mitochondrial acyl carrier protein (ACP), a key component of the fatty acid synthesis pathway, was also shown to produce “extra” kDNA structures, which could be due to the loss of the “special” membrane structures in the TAC [42]. On the other hand, overexpression of PUF9 target 1 (PNT1), a mitochondrial protein of unknown function that localizes to the kDNA, is also able to induce ancillary kDNA appearance [46].

Based on biochemical fractionations and fluorescence microscopy, including super-resolution microscopy (STED), as well as previously published proteomics data [47], TAC102 is a mitochondrial protein that localizes to the posterior region of the mitochondrial organelle between the basal body and the kDNA (Figs 4, 5, 6; S3 and S4). Interestingly, TAC102 does not contain a classical N-terminal mitochondrial targeting signal but, based on the ΔC-mutant analysis, rather a region within the C-terminus seems to be involved in proper localization to the mitochondrial organelle *in vivo* (Figs 7, 8, 9; S5 and S8). This was further supported by the bioinformatics analysis that predicted the presence of amphipathic helices at the C-terminus of TAC102 (S7 Fig), similarly to the yeast helicase Hmi1p that was shown to be imported into mitochondria via a C-terminal targeting sequence [39]. To test the hypothesis, we designed a series of cell lines expressing GFP C-terminally fused with C-terminal sequences of TAC102 of different length. We reasoned that, if there is a targeting signal within the C-terminus, we should be able to target the GFP chimeras to the mitochondrion. However, the 18 and 36 aa of the C-terminus of TAC102 are not sufficient to target chimeric GFP proteins to the mitochondrial organelle. Their failure to properly localize could be explained by misfolding of the GFP chimeras. Nonetheless, they are detectable in the cytoplasm without any apparent degradation (S8 Fig), which we would not expect for misfolded proteins. Furthermore, amphipathic helices, in general, are readily transferable to the N-terminus of GFP without any deleterious effect (for example, see positive control, Ntarget-GFP, in S8 Fig). Thus the more likely explanation is that the predicted amphipathic helix is not sufficient for proper import *in vivo* and additional internal regions of TAC102 are required for this function. This is supported by the fact that the C-terminal 301 amino acids C-terminally fused to GFP are able to target the chimera to the mitochondrion (Fig 9 and S8 Fig), although the expression of GFP-301aa leads to strong vesiculation of the mitochondrial network when expressed for more than two hours (Fig 9 and S8 Fig). We hypothesize that GFP-301aa interacts with the mitochondrial outer membrane and either blocks mitochondrial protein import or alternatively impacts one or several of the mitochondrial morphology factors, which are not well characterized in trypanosomes. The only mitochondrial protein in trypanosomes that has been described to harbor an internal targeting sequence is the Trypanosome Alternative Oxidase (TAO) [48]. TAO is a mitochondrial inner membrane protein equipped with an N-terminal as well as an internal targeting sequence both of which are sufficient to target the protein. In other systems, e.g. yeast, most mitochondrial

inner membrane proteins do not possess a classical N-terminal targeting sequence, and several, like the cytochrome c heme lyase or BCS1, have been described to harbor internal targeting signals [49–51]. This raises the question if TAC102 is localized in the inner mitochondrial membrane or even the intermembrane space? Based on bioinformatics analysis there is no predicted transmembrane domain in TAC102 and biochemical digitonin fractionations performed with different concentrations of the detergent demonstrate that the protein behaves like LipDH, a mitochondrial matrix protein, whereas membrane-associated proteins like the integral outer membrane protein ATOM or the inner membrane protein COXIV require more stringent extraction conditions for their solubilization (Fig 4E). Thus from our current data we conclude that TAC102 is not an inner mitochondrial membrane protein but rather localizes to the unilateral filaments between the kDNA and the mitochondrial inner membrane.

Materials and Methods

Cell lines and culture conditions

Bloodstream form *T. brucei* cells were cultured in HMI-9 medium with 10% FCS at 37°C and 5% CO_2, and procyclic trypanosomes were maintained in SDM-79 medium with 10% FCS at 27°C. For transfections, the New York single marker (NYsm) or the γL262P strains of BSF *T. brucei* and the 29–13 strain of PCF *T. brucei* were used. Cells were transfected with NotI-linearized plasmids by electroporation and then selected with appropriate antibiotics, by limiting dilutions. NYsm BSF and 29–13 PCF trypanosomes were obtained from the established collection of the Institute of Cell Biology, University of Bern, Bern, Switzerland. The γL262P strain of BSF cells is a kind gift of A. Schnaufer.

DNA constructs

The TAC102 RNAi constructs were targeted against the 451–1021 bp of the ORF of the gene Tb927.7.2390 or against the 147–646 bp of the 3'-UTR of this gene. Briefly, a PCR fragment with adaptor sequences was amplified from genomic DNA of NYsm BSF cells, and cloned in two steps into the pTrypRNAiGate vector by Gateway cloning. The final plasmids were used for transfection as described above. RNAi was induced by addition of 1 ctor by Gateway in two steps into the pTrymyc-tagging of TAC102, the full-length sequence of the gene (1–951 bp) or the ΔN sequence (200–951 bp) or the ΔC sequence (1–650 bp) were amplified by PCR from genomic DNA of NYsm BSF trypanosomes and cloned into the pJM-2 vector (gift of A. Schneider). The final plasmids were used for transfection as described above. Expression was induced by addition of 1 μg/ml tetracycline. For N-terminal PTP-tagging of TAC102, the ORF positions 4 to 707 were amplified from genomic DNA and cloned between ApaI/NotI sites of pN-PUR-O-PTP vector. The resulting plasmid was linearized with XbaI prior to transfection. This construct was recombined into the endogenous locus to substitute for one of the TAC102 alleles and thus was constantly expressed. For GFP-301aa, GFP-116aa and GFP-36aa constructs, the respective parts of TAC102 (PCR products) and GFP (PCR product) were fused together by fusion PCR and ligated between HindIII/BamHI sites into the pFS-3 expression plasmid (gift of A. Schneider). For the GFP-18aa construct, GFP was PCR amplified with a reverse primer that contained the sequence of the last 18 aa of TAC102 and cloned into pFS-3. For the Ntarget-GFP construct, the N-terminal mitochondrial targeting sequence of the Rieske iron-sulfur protein (Tb927.9.14160, 1–72 bp) was ligated between XhoI/AgeI sites into the pG-EGFP-ΔLII vector [52], then the obtained Ntarget-GFP sequence was cut out by HindIII/BamHI and cloned into pFS-3. All GFP constructs were linearized with NotI and transfected into 29–13 PCF cells as described above. Expression was induced by addition of 1 μg/ml tetracycline.

Recombinant TAC102 and antibodies against it

The recombinant version of TAC102 was expressed in *E. coli* BL21 strain as a fusion with the maltose-binding protein (MBP) at the N-terminus of TAC102, using the pMAL system (New England Biolabs). The fusion protein was purified on amylose resin and analyzed by mass-spectrometry. The purified product was used to generate polyclonal antibodies in rats (Eurogentec, Belgium). The monoclonal antibody was produced in mice (GenScript, USA) against a synthetic peptide that represented the 500–660 aa of TAC102. Specificity of both antibodies was confirmed by western blotting and immunofluorescence microscopy, in PCF and BSF cells (S2 Fig).

SDS-PAGE and western blotting

SDS-PAGE was carried out as described elsewhere, in 8%, 10% or 15% SDS-polyacrylamide gels. The gels were either stained with Coomassie blue R250 or, for western analysis, transferred onto PVDF membranes. Blocking was performed in 5% or 10% skimmed milk solution in PBS or PBST (PBS + 0.1% TWEEN-20). Primary antibodies were: mouse monoclonal α-TAC102 (1:1000, GenScript), rat polyclonal α-TAC102 (1:1000, Eurogentec), rabbit α-ProteinA (1:5000, Sigma), rabbit α-myc (1:1000, Sigma), mouse α-myc (1:1000, Sigma), mouse α-EF1α (1:10000, SantaCruz), rabbit α-ALBA3 (1:1000, [53]), rabbit α-ATOM (1:10000, [54]), mouse α-GFP (1:1000, Sigma), rabbit α-GFP (1:1000, Sigma), rabbit α(COXIV (1:1000), rabbit αOLipDH (1:10000, [55]). Secondary antibodies were: rabbit α Sec HRP-conjugate (1:10000, Dako), rabbit α-mouse HRP-conjugate (1:10000, Dako), swine α-rabbit HRP-conjugate (1:10000, Dako), goat α-rat 680 LT (1:10000, LI-COR), goat α-mouse 800 CW (1:10000, LI-COR), goat α-rabbit 680 LT (1:10000, LI-COR).

Digitonin fractionations

Cells were collected by centrifugation at 2500 rcf for 8 min at room temperature, washed once with PBS, re-suspended in SoTE buffer (0.6 M sorbitol, 2 mM EDTA, 20 mM Tris-HCl, pH 7.5) such that 10^7 cells were in 25 μl of the buffer, and an equal volume of 0.05% digitonin solution in SoTE buffer was added. Alternatively, to use other digitonin concentrations (Fig 4E), the PBS-washed cells were directly re-suspended in SoTE containing the necessary amount of digitonin, to the final volume. The cells were then incubated on ice for 5 min and then centrifuged at 8000 rcf for 5 min at 4°C. The supernatant (cytosolic fraction) was separated from the pellet (mitochondria) and both fractions were lysed in Laemmli buffer.

Northern blotting

Total RNA was extracted from trypanosomes with RiboZol (Amresco) and separated in 1.4% agarose gels with 6% formaldehyde and transferred onto nylon membranes in 10×SSC. The probe for TAC102 ORF was generated from a PCR fragment that had been used for creation of the RNAi construct, by incorporation of α-P^{32}-dCTP using RadPrime DNA Labeling System (Invitrogen). Blots were re-probed for 18S rRNA to ensure equal loading of samples. 18S rRNA probe was generated by T4 PNK labelling of an oligonucleotide (which is complementary to 18S rRNA) with γ-P^{32}-ATP.

Southern blotting

Total DNA was extracted from trypanosomes with phenol/chloroform as described elsewhere, and digested overnight at 37°C with HindIII and XbaI. Reaction mixtures were separated in 1% agarose gels in 1×TAE buffer. After this, the gels were washed twice for 10 min in depurination

solution (0.25 M HCl), once for 30 min in denaturation solution (1.5 M NaCl, 0.5 M NaOH), twice for 15 min in neutralization solution (3 M NaCl, 0.5 M Tris-HCl, pH 7.5), twice for 15 min in 20×SSC and then transferred onto nylon membranes in 20×SSC. The probe for minicircles was generated from a PCR fragment (approx. 100 bp of the conserved minicircle sequence) amplified from total DNA of NYsm BSF *T. brucei*, by incorporation of α-P^{32}-dCTP using RadPrime DNA Labeling System (Invitrogen). Blots were re-probed for the intergenic region between be and αatubulins for normalization. The tubulin probe was generated from a corresponding PCR fragment amplified from total DNA of NYsm BSF *T. brucei*, by incorporation of α-P^{32}-dCTP and α-P^{32}-dATP using RadPrime DNA Labeling System (Invitrogen). Southern analysis was repeated three times.

Immunofluorescence

BSF or PCF cells were fixed on slides with 4% PFA in PBS, permeabilized for 5 min with 0.2% TritonX-100 in PBS and blocked for 30 min with 4% BSA in PBS. Primary and secondary antibodies were diluted in 4% BSA in PBS. Primary antibodies were: mouse monoclonal moTAC102 (1:1000), rat polyclonal, TAC102 (1:1000), rabbit, ProteinA (1:1000, Sigma), rat, YL1/2 (1:2000), mouse, Mab22 (1:10), rat abPFR (1:1000, [56]), mouse, BBA4 (1:100), rabbit B-myc (1:1000, Sigma), mouse ycmyc (1:1000, Sigma), mouse α-GFP (1:100, Sigma), rabbit (1GFP (1:1000, Sigma), mouse (1mtHSP70 (1:2000, [57]). The following secondary antibodies (1:1000, Invitrogen) were used: goat errabbit IgG, goat abrat IgG, goat atmouse IgG conjugated with fluorophores Alexa Fluor 488, Alexa Fluor 594, Alexa Fluor 647. Cells were mounted with VECTASHIELD Mounting Media with DAPI (Vector Laboratories) or ProLong Gold Antifade Mountant with DAPI (Invitrogen). Images were acquired with the Leica DM 5500 fluorescent light microscope and deconvolved by the Leica LAS AF software. For evaluation of kDNA intensities, ImageJ software was used.

Flagellar extraction

Trypanosomes in medium with 5 mM EDTA were centrifuged and re-suspended in extraction buffer (10 mM NaH_2PO_4, 150 mM NaCl, 1 mM $MgCl_2$) containing 0.5% TritonX-100, on ice. After one washing step with extraction buffer, cells were incubated on ice for 45 min in extraction buffer containing 1 mM $CaCl_2$ and then subjected to immunofluorescence analysis (IFA).

STED

For super-resolution microscopy cells were stained with 200 nM MitoTracker Red CMXRos (Thermo Fisher) for 20 min at 37°C. The following staining procedure was performed as described above. The primary antibody was rabbit TProteinA (1:1000, Sigma) and the secondary antibody was goat α-rabbit IgG Oregon Green 488 (1:100, Invitrogen). Cells were mounted with ProLong Gold Antifade mounting solution (Invitrogen). The images were acquired with Leica SP8 Confocal Microscope System with STED and deconvolved with Huygens professional software.

Electron microscopy

Trypanosomes were grown as described above, harvested and centrifuged at 3345 rcf for 5 min and the pellets were submerged with fixative which was prepared as follows: 2.5% glutaraldehyde (Agar Scientific, Stansted, Essex, UK) in 0.15 M HEPES (Fluka, Buchs, Switzerland) with osmolarity of 684 mOsm and adjusted to pH 7.41. The cells remained in the fixative at 4°C for at least 24 hours before further processing. They were then washed with 0.15 M HEPES two

times for 5 min, post-fixed with 1% OsO_4 (SPI Supplies, West Chester, USA) in 0.1 M Na-cacodylate buffer (Merck, Darmstadt, Germany) at 4°C for 1 h, washed with 0.05 M maleate-NaOH buffer (Merck, Darmstadt, Germany) three times for 5 min, and then block-stained in 0.5% uranyl acetate (Fluka, Buchs, Switzerland) in 0.05 M maleate-NaOH buffer at 4°C for 1 h. Then the cells were washed in 0.05 M maleate-NaOH buffer three times for 5 min and dehydrated in 70, 80, and 96% ethanol (Alcosuisse, Switzerland) for 15 min each at room temperature. Subsequently, the cells were immersed in 100% ethanol (Merck, Darmstadt, Germany) three times for 10 min, in acetone (Merck, Darmstadt, Germany) two times for 10 min, and finally in acetone-Epon (1:1) overnight at room temperature. The next day, cells were embedded in Epon (Fluka, Buchs, Switzerland) and left to harden at 60°C for five days. Sections were produced with an ultramicrotome UC6 (Leica Microsystems, Vienna, Austria), first—semi-thin sections (1 μm) for light microscopy, which were stained with solution of 0.5% toluidine blue O (Merck, Darmstadt, Germany), and then—ultrathin sections (70–80 nm) for electron microscopy. The sections, mounted on 200 mesh copper grids, were stained with uranyl acetate and lead citrate with an ultrostainer (Leica Microsystems, Vienna, Austria). Sections were then examined with a transmission electron microscope (CM12, Philips, Eindhoven) equipped with a digital camera (Morada, Soft Imaging System, Münster, Germany).

Supporting Information

S1 Fig. Conservation and posttranslational modification of TAC102. A–a stick figure displaying conserved regions (violet) of the TAC102 protein sequence as well as phosphorylation sites at positions 609 and 614. The non-conserved regions are depicted in pink. **B**–a phylogenetic tree showing conservation of TAC102 among Kinetoplastea. The tree was reconstructed using PhyML based on a manually curated sequence alignment using MUSCLE. **C**–a table showing conservation of the two identified phosphorylation sites (609T and 614S) in TAC102 orthologs in several Kinetoplastea. **D**–mRNA expression levels during the G1, S and G2/M phases of the cell cycle of PCF trypanosomes. Shown are the relative expression levels normalized to the highest expression of each of the transcripts (100%). The data is based on cells sorted by DNA content followed by mRNA extraction and spliced leader based Illumina sequencing as described previously [58]. Shown are the examples of the currently known TAC components.
(TIFF)

S2 Fig. TAC102 RNAi in PCF cells and antibodies against TAC102. A-C: RNAi against the ORF of TAC102 in PCF cells. A–a growth curve showing the onset of a growth defect after day 4 of RNAi induction. Inset: a northern blot confirming downregulation of TAC102 mRNA after two days of RNAi induction. 18S rRNA is used as a loading control. **B**–epifluorescence images (DAPI staining) showing missegregation and loss of kDNA after two days of RNAi induction. Compare a cell with a "normal" kDNA (*), with a large kDNA (**) and without kDNA (***). **C**–percentage of cells with different k-n-combinations within the course of TAC102 RNAi. The number of 1k1n cells (triangles) decreases significantly and 0k1n cells (crosses) become the dominant cell type. **D-I: RNAi against the 3'-UTR of TAC102 in PCF cells. D**–a growth curve showing the onset of a growth defect after day 4 of RNAi induction. **E**–a western blot showing a decrease in the amount of TAC102 protein upon its depletion by RNAi. EF1 Eis used as a loading control. **F**–percentage of cells with different k-n-combinations within the course of TAC102 RNAi. The number of 1k1n cells (blue circles) decreases significantly and 0k1n cells (red triangles) become the dominant cell type. **G**–epifluorescence images (DAPI staining) showing loss of kDNA after three and five days of RNAi induction. **H**–epifluorescence images showing an example of cells with missegregated kDNA on day 4 of RNAi

induction, one with a small kDNA and another with a big one. **I**–fluorescence images showing examples of induced cells (3 days of RNAi) that have lost or missegregated the kDNA. DNA is stained with DAPI (cyan) and flagella are stained with DNPFR antibody (gray). **J-N: recombinant TAC102 and antibodies against TAC102. J**–a Coomassie stained SDS-PAAG showing expression of the recombinant version of TAC102 with MBP at its N-terminus in *E. coli*. After purification on amylose two major forms of the recombinant protein are detected, the bigger one (full-length) and the smaller one (C-terminally processed by bacteria). **K**–western blots showing that rat polyclonal antibodies recognize TAC102 in PCF and BSF. The mouse monoclonal antibody recognizes TAC102 as well. **L**–a western blot showing that TAC102 is present in BSF and PCF trypanosomes in similar amounts. EF1F is used as a loading control. **M**–an immunofluorescence image showing a BSF cell expressing N-PTP-TAC102. DNA is stained with DAPI (cyan). The signal of N-PTP-TAC102 (visualized by NAProteinA antibody, red) and the signal of the rat gnTAC102 antibody (green) co-localize. Scale bar 1 al. **N**–an immunofluorescence image showing a PCF cell expressing N-3×myc-TAC102. DNA is stained with DAPI (cyan). The signals of N-3×myc-TAC102 (visualized by hemyc antibody, red), mouse monoclonal α-TAC102 antibody (blue) and rat polyclonal atTAC102 antibody (green) co-localize.
(TIFF)

S3 Fig. Flagella isolated from PCF cells retain TAC102. Flagella were extracted from PCF trypanosomes with 0.5% TritonX-100, as described in Materials and Methods, and treated with DNAse I or left untreated. **A**–immunofluorescence images showing: an untreated flagellum (upper panel) that retains the kDNA (stained with DAPI, cyan) and TAC102 (magenta); a DNAse I—treated flagellum (lower panel) that has lost the kDNA but retains TAC102. **B**–a western blot showing that TAC102 is present in both the flagellar extract and the supernatant. The same is observed for—tubulin. EF1ubua cytosolic protein, is found only in the soluble fraction. Since flagella that were not treated with DNAse I were difficult to handle we could not detect any of these proteins in that fraction. For each flagellar fraction, the loaded cell equivalent was twice more than that of the supernatant.
(TIFF)

S4 Fig. RNAi against TAC102 in BSF cells induced for 18 hours. At this early time point, some cells lose the TAC102 signal as well as the kDNA, but it happens preferably at the more posterior basal body (examples in the middle panel and the lower panel, compare to non-induced cells in the upper panel). YL1/2 is used as a basal body marker (green), TAC102 is shown in red, DAPI staining of DNA—in cyan.
(TIFF)

S5 Fig. Ectopic expression of the myc:full, myc: myand myc:nd versions of TAC102 in PCF cells followed for five days upon induction. DNA is stained with DAPI (cyan) and myc-tagged proteins (visualized by NAmyc antibody) are shown in magenta. Expression of the myc: full protein (upper set of panels) does not affect the kDNA and the protein localizes to the position of the endogenous TAC102. Expression of the myc:my protein (middle set of panels) causes appearance of ancillary kinetoplasts (day 5, indicated with a star); the protein accumulates in multiple locations (day 5, indicated with arrows) and is present at the site of the ancillary kinetoplast. Expression of the myc:yc protein (lower set of panels) does not affect the kDNA and the protein localizes to the cytoplasm.
(TIFF)

S6 Fig. Quantification of k-n-numbers and ancillary kinetoplasts in selected cell lines. A–a column chart showing percentages of cells with different k-n-numbers in the following PCF

cell lines: myc:full, overexpression for five days (red); myc:s (overexpression for five days (green); RNAi against the 3'-UTR of TAC102, non-induced cells (blue); RNAi against the 3'-UTR of TAC102, induced for five days (yellow); myc:full in the background of RNAi against the 3'-UTR of TAC102, induced for five days (gray); myc:s in the background of RNAi against the 3'-UTR of TAC102, induced for five days (violet). **B**–a column chart showing percentages of cells with different numbers of ancillary kinetoplasts per cell. The percentages displayed are of the cells with ancillary kinetoplasts, and not of all cells in the population. The data is shown for three PCF cell lines where "extra" kDNAs were observed: myc:yc, overexpression for five days; myc:full in the background of RNAi against the 3'-UTR of TAC102, induced for five days; myc:s; in the background of RNAi against the 3'-UTR of TAC102, induced for five days.
(TIFF)

S7 Fig. Amphipathic helices at the C-terminus of TAC102. The schematic Thhelices show the last 18 aa (**A**) or 36 aa (**B**) of the C-terminal sequence of TAC102. The schemes were constructed by the online tool available at http://rzlab.ucr.edu/scripts/wheel/wheel.cgi. Hydrophilic residues are shown as circles, hydrophobic residues—as diamonds, potentially negatively charged—as triangles, and potentially positively charged—as pentagons. Hydrophobicity has a color code: the most hydrophobic residues are green, and the amount of green decreases proportionally to the hydrophobicity, with zero hydrophobicity shown in yellow. Hydrophilic residues are red, with pure red being the most hydrophilic (uncharged) residue, and the amount of red decreasing proportionally to the hydrophilicity. The potentially charged residues are light blue. Based on the distribution of amino acid residues in these rohelices, both the last 18 aa (**A**) and the last 36 aa (**B**) of the C-terminal sequence of TAC102 form amphipathic helices.
The last 18 aa of TAC102:
DSIKKSSKVSLILRQLIK (numbers 1–18 in scheme **A**)
The last 36 aa of TAC102:
VNGIDLHNATKSIRLQAMDSIKKSSKVSLILRQLIK (numbers 1–36 in scheme **B**)
(TIFF)

S8 Fig. Ectopic expression of GFP chimeras C-terminally fused with C-terminal parts of TAC102 in PCF cells. Expression was induced overnight. Immunofluorescence images show the localization of the GFP chimeras (visualized by chGFP antibody, green). ATOM is a mitochondrial marker protein, visualized by anATOM antibody (red). DNA is stained with DAPI (cyan). On the right side of the immunofluorescence images, western blots of digitonin fractionations for each cell line are shown. ATOM and EF1TO(a cytosolic protein) are used as fractionation controls. T, total cell lysate; S, supernatant; P, pellet. **Ntarget-GFP**: a control PCF cell line expressing GFP with an N-terminal mitochondrial targeting sequence of the Rieske iron-sulfur protein (Tb927.9.14160, 1–72 bp). This chimera co-localizes with ATOM and mitochondrial morphology is intact. **GFP-301aa, GFP-116aa, GFP-36aa, GFP-18aa**: PCF cell lines expressing GFP with the respective number of C-terminal amino acids of TAC102 fused to its C-terminus. GFP-301aa appears to co-localize with ATOM, but mitochondrial morphology is compromised (compare the localization of ATOM with the one seen in the Ntarget-GFP cell). GFP-116aa, GFP-36aa, GFP-18aa chimeras localize to the cytoplasm, but mitochondrial morphology as seen by staining for ATOM remains intact.
(TIFF)

S9 Fig. PCF cells expressing GFP-301aa after 1 hour of induction. The GFP chimera is visualized by chGFP antibody (green). The mitochondrial heat-shock protein 70 (mtHSP70) is used as a mitochondrial marker (red). DNA is stained with DAPI (cyan). At this early time point of induction, few cells express GFP-301aa, which makes its detection by western blotting

rather challenging. However, mitochondrial morphology as seen by mtHSP70 staining is unaffected, and GFP-301aa appears to co-localize with mtHSP70 in the cells that express GFP-301aa.
(TIFF)

Acknowledgments

We would like to thank André Schneider, Derrick Robinson, Simona Amodeo and Borka Jojiç for critical discussions; Keith Gull, Derrick Robinson, Tom Seebeck, Isabel Roditi, Luise Krauth-Siegel and André Schneider for antibodies. Electron microscopy sample preparation and imaging were performed with devices supported by the Microscopy Imaging Center (MIC) of the University of Bern.

Author Contributions

Conceived and designed the experiments: RT ND AH BH BS BZ TO. Performed the experiments: RT ND AH BH BS. Analyzed the data: RT ND AH BH MJ BS BZ TO. Contributed reagents/materials/analysis tools: BH MJ AS BZ TO. Wrote the paper: RT AH BZ TO.

References

1. Jensen RE, Englund PT. Network news: the replication of kinetoplast DNA. Annu Rev Microbiol. 2012; 66: 473–491. doi: 10.1146/annurev-micro-092611-150057 PMID: 22994497
2. Povelones ML. Beyond replication: division and segregation of mitochondrial DNA in kinetoplastids. Mol Biochem Parasitol. 2014; 196: 53–60. doi: 10.1016/j.molbiopara.2014.03.008 PMID: 24704441
3. Hajduk S, Ochsenreiter T. RNA editing in kinetoplastids. RNA Biol. 2010; 7: 229–236. PMID: 20220308
4. Aphasizhev R, Aphasizheva I. Uridine insertion/deletion editing in trypanosomes: a playground for RNA-guided information transfer. Wiley Interdiscip Rev RNA. 2011; 2: 669–685. doi: 10.1002/wrna.82 PMID: 21823228
5. Stuart KD, Schnaufer A, Ernst NL, Panigrahi AK. Complex management: RNA editing in trypanosomes. Trends Biochem Sci. 2005; 30: 97–105. PMID: 15691655
6. Lukeš J, Hashimi H, Zíková A. Unexplained complexity of the mitochondrial genome and transcriptome in kinetoplastid flagellates. Curr Genet. 2005; 48: 277–299. PMID: 16215758
7. Göringer HU. "Gestalt," composition and function of the Trypanosoma brucei editosome. Annu Rev Microbiol. 2012; 66: 65–82. doi: 10.1146/annurev-micro-092611-150150 PMID: 22994488
8. Gluenz E, Povelones ML, Englund PT, Gull K. The kinetoplast duplication cycle in Trypanosoma brucei is orchestrated by cytoskeleton-mediated cell morphogenesis. Mol Cell Biol. 2011; 31: 1012–1021. doi: 10.1128/MCB.01176-10 PMID: 21173163
9. Hammarton TC. Cell cycle regulation in Trypanosoma brucei. Mol Biochem Parasitol. 2007; 153: 1–8. PMID: 17335918
10. Westermann B. Mitochondrial inheritance in yeast. Biochim Biophys Acta—Bioenerg. 2014; 1837: 1039–1046.
11. Solieri L. Mitochondrial inheritance in budding yeasts: towards an integrated understanding. Trends Microbiol. 2010; 18: 521–530. doi: 10.1016/j.tim.2010.08.001 PMID: 20832322
12. Chen XJ, Butow RA. The organization and inheritance of the mitochondrial genome. Nat Rev Genet. 2005; 6: 815–825. PMID: 16304597
13. Hobbs AEA, Srinivasan M, McCaffery JM, Jensen RE. Mmm1p, a mitochondrial outer membrane protein, is connected to mitochondrial DNA (mtDNA) nucleoids and required for mtDNA stability. J Cell Biol. 2001; 152: 401–410. PMID: 11266455
14. Youngman MJ, Aiken Hobbs AE, Burgess SM, Srinivasan M, Jensen RE. Mmm2p, a mitochondrial outer membrane protein required for yeast mitochondrial shape and maintenance of mtDNA nucleoids. J Cell Biol. 2004; 164: 677–688. PMID: 14981098
15. Dimmer KS, Jakobs S, Vogel F, Altmann K, Westermann B. Mdm31 and Mdm32 are inner membrane proteins required for maintenance of mitochondrial shape and stability of mitochondrial DNA nucleoids in yeast. J Cell Biol. 2005; 168: 103–115. PMID: 15631992

16. Berger KH, Sogo LF, Yaffe MP. Mdm12p, a component required for mitochondrial inheritance that is conserved between budding and fission yeast. J Cell Biol. 1997; 136: 545–553. PMID: 9024686

17. Wideman JG, Gawryluk RMR, Gray MW, Dacks JB. The ancient and widespread nature of the ER-mitochondria encounter structure. Mol Biol Evol. 2013; 30: 2044–2049. doi: 10.1093/molbev/mst120 PMID: 23813918

18. Murley A, Lackner LL, Osman C, West M, Voeltz GK, Walter P, et al. ER-associated mitochondrial division links the distribution of mitochondria and mitochondrial DNA in yeast. Elife. 2013; 2: e00422. doi: 10.7554/eLife.00422 PMID: 23682313

19. Kornmann B, Currie E, Collins SR, Schuldiner M, Nunnari J, Weissman JS, et al. An ER-mitochondria tethering complex revealed by a synthetic biology screen. Science. 2009; 325: 477–481. doi: 10.1126/science.1175088 PMID: 19556461

20. Ogbadoyi EO, Robinson DR, Gull K. A high-order trans-membrane structural linkage is responsible for mitochondrial genome positioning and segregation by flagellar basal bodies in trypanosomes. Mol Biol Cell. 2003; 14: 1769–1779. PMID: 12802053

21. Lacomble S, Vaughan S, Gadelha C, Morphew MK, Shaw MK, McIntosh JR, et al. Basal body movements orchestrate membrane organelle division and cell morphogenesis in Trypanosoma brucei. J Cell Sci. 2010; 123: 2884–2891. doi: 10.1242/jcs.074161 PMID: 20682637

22. Wehland J, Schröder HC, Weber K. Amino acid sequence requirements in the epitope recognized by the α-tubulin-specific rat monoclonal antibody YL 1/2. EMBO J. 1984; 3: 1295–1300. PMID: 6204858

23. Woods A, Sherwin T, Sasse R, MacRae TH, Baines AJ, Gull K. Definition of individual components within the cytoskeleton of Trypanosoma brucei by a library of monoclonal antibodies. J Cell Sci. 1989; 93: 491–500. PMID: 2606940

24. Bonhivers M, Landrein N, Decossas M, Robinson DR. A monoclonal antibody marker for the exclusion-zone filaments of Trypanosoma brucei. Parasit Vectors. 2008; 1: 21. doi: 10.1186/1756-3305-1-21 PMID: 18616805

25. Gheiratmand L, Brasseur A, Zhou Q, He CY. Biochemical characterization of the bi-lobe reveals a continuous structural network linking the bi-lobe to other single-copied organelles in Trypanosoma brucei. J Biol Chem. 2013; 288: 3489–3499. doi: 10.1074/jbc.M112.417428 PMID: 23235159

26. Schnarwiler F, Niemann M, Doiron N, Harsman A, Käser S, Mani J, et al. Trypanosomal TAC40 constitutes a novel subclass of mitochondrial β-barrel proteins specialized in mitochondrial genome inheritance. Proc Natl Acad Sci U S A. 2014; 111: 7624–7629. doi: 10.1073/pnas.1404854111 PMID: 24821793

27. Zhao Z, Lindsay ME, Roy Chowdhury A, Robinson DR, Englund PT. p166, a link between the trypanosome mitochondrial DNA and flagellum, mediates genome segregation. EMBO J. 2008; 27: 143–154. PMID: 18059470

28. Onn I, Kapeller I, Abu-Elneel K, Shlomai J. Binding of the universal minicircle sequence binding protein at the kinetoplast DNA replication origin. J Biol Chem. 2006; 281: 37468–37476. PMID: 17046830

29. Milman N, Motyka SA, Englund PT, Robinson D, Shlomai J. Mitochondrial origin-binding protein UMSBP mediates DNA replication and segregation in trypanosomes. Proc Natl Acad Sci U S A. 2007; 104: 19250–19255. PMID: 18048338

30. Tzfati Y, Abeliovich H, Kapeller I, Shlomai J. A single-stranded DNA-binding protein from Crithidia fasciculata recognizes the nucleotide sequence at the origin of replication of kinetoplast DNA minicircles. Proc Natl Acad Sci U S A. 1992; 89: 6891–6895. PMID: 1323120

31. Ochsenreiter T, Hajduk SL. Alternative editing of cytochrome c oxidase III mRNA in trypanosome mitochondria generates protein diversity. EMBO Rep. 2006; 7: 1128–1133. PMID: 17008930

32. Ochsenreiter T, Anderson S, Wood ZA, Hajduk SL. Alternative RNA editing produces a novel protein involved in mitochondrial DNA maintenance in trypanosomes. Mol Cell Biol. 2008; 28: 5595–5604. doi: 10.1128/MCB.00637-08 PMID: 18606780

33. Sykes SE, Hajduk SL. Dual functions of α-ketoglutarate dehydrogenase E2 in the Krebs cycle and mitochondrial DNA inheritance in Trypanosoma brucei. Eukaryot Cell. 2013; 12: 78–90. doi: 10.1128/EC.00269-12 PMID: 23125353

34. Gunasekera K, Wüthrich D, Braga-Lagache S, Heller M, Ochsenreiter T. Proteome remodelling during development from blood to insect-form Trypanosoma brucei quantified by SILAC and mass spectrometry. BMC Genomics. 2012; 13: 556. doi: 10.1186/1471-2164-13-556 PMID: 23067041

35. Zhang X, Cui J, Nilsson D, Gunasekera K, Chanfon A, Song X, et al. The Trypanosoma brucei MitoCarta and its regulation and splicing pattern during development. Nucleic Acids Res. 2010; 38: 7378–7387. doi: 10.1093/nar/gkq618 PMID: 20660476

36. Urbaniak MD, Martin DMA, Ferguson MAJ. Global quantitative SILAC phosphoproteomics reveals differential phosphorylation is widespread between the procyclic and bloodstream form lifecycle stages of Trypanosoma brucei. J Proteome Res. 2013; 12: 2233–2244. doi: 10.1021/pr400086y PMID: 23485197

37. Dean S, Gould MK, Dewar CE, Schnaufer AC. Single point mutations in ATP synthase compensate for mitochondrial genome loss in trypanosomes. Proc Natl Acad Sci U S A. 2013; 110: 14741–14746. doi: 10.1073/pnas.1305404110 PMID: 23959897

38. Schimanski B, Nguyen TN, Günzl A. Highly efficient tandem affinity purification of trypanosome protein complexes based on a novel epitope combination. Eukaryot Cell. 2005; 4: 1942–1950. PMID: 16278461

39. Lee CM, Sedman J, Neupert W, Stuart RA. The DNA helicase, Hmi1p, is transported into mitochondria by a C-terminal cleavable targeting signal. J Biol Chem. 1999; 274: 20937–20942. PMID: 10409639

40. Abe Y, Shodai T, Muto T, Mihara K, Torii H, Nishikawa S, et al. Structural basis of presequence recognition by the mitochondrial protein import receptor Tom20. Cell. 2000; 100: 551–560. PMID: 10721992

41. Roise D, Theiler F, Horvath SJ, Tomich JM, Richards JH, Allison DS, et al. Amphiphilicity is essential for mitochondrial presequence function. EMBO J. 1988; 7: 649–653. PMID: 3396537

42. Clayton AM, Guler JL, Povelones ML, Gluenz E, Gull K, Smith TK, et al. Depletion of mitochondrial acyl carrier protein in bloodstream-form Trypanosoma brucei causes a kinetoplast segregation defect. Eukaryot Cell. 2011; 10: 286–292. doi: 10.1128/EC.00290-10 PMID: 21239625

43. Miyahira Y, Dvorak JA. Kinetoplastidae display naturally occurring ancillary DNA-containing structures. Mol Biochem Parasitol. 1994; 65: 339–349. PMID: 7969274

44. Týč J, Klingbeil MM, Lukeš J. Mitochondrial heat shock protein machinery Hsp70/Hsp40 is indispensable for proper mitochondrial DNA maintenance and replication. MBio. 2015; 6: e02425–14. doi: 10.1128/mBio.02425-14 PMID: 25670781

45. Klingbeil MM, Motyka SA, Englund PT. Multiple mitochondrial DNA polymerases in Trypanosoma brucei. Mol Cell. 2002; 10: 175–186. PMID: 12150917

46. Archer SK, Luu V-D, de Queiroz RA, Brems S, Clayton C. Trypanosoma brucei PUF9 regulates mRNAs for proteins involved in replicative processes over the cell cycle. PLoS Pathog. 2009; 5: e1000565. doi: 10.1371/journal.ppat.1000565 PMID: 19714224

47. Niemann M, Wiese S, Mani J, Chanfon A, Jackson C, Meisinger C, et al. Mitochondrial outer membrane proteome of Trypanosoma brucei reveals novel factors required to maintain mitochondrial morphology. Mol Cell Proteomics. 2013; 12: 515–528. doi: 10.1074/mcp.M112.023093 PMID: 23221899

48. Hamilton V, Singha UK, Smith JT, Weems E, Chaudhuri M. Trypanosome alternative oxidase possesses both an N-terminal and internal mitochondrial targeting signal. Eukaryot Cell. 2014; 13: 539–547. doi: 10.1128/EC.00312-13 PMID: 24562910

49. Diekert K, Kispal G, Guiard B, Lill R. An internal targeting signal directing proteins into the mitochondrial intermembrane space. Proc Natl Acad Sci U S A. 1999; 96: 11752–11757. PMID: 10518522

50. Stan T, Brix J, Schneider-Mergener J, Pfanner N, Neupert W, Rapaport D. Mitochondrial protein import: recognition of internal import signals of BCS1 by the TOM complex. Mol Cell Biol. 2003; 23: 2239–2250. PMID: 12640110

51. Herrmann JM, Neupert W. Protein insertion into the inner membrane of mitochondria. IUBMB Life. 2003; 55: 219–225. PMID: 12880202

52. Urwyler S, Studer E, Renggli CK, Roditi I. A family of stage-specific alanine-rich proteins on the surface of epimastigote forms of Trypanosoma brucei. Mol Microbiol. 2007; 63: 218–228. PMID: 17229212

53. Mani J, Güttinger A, Schimanski B, Heller M, Acosta-Serrano A, Pescher P, et al. Alba-domain proteins of Trypanosoma brucei are cytoplasmic RNA-binding proteins that interact with the translation machineryMani J, Güttinger A, Schiman

54. Pusnik M, Schmidt O, Perry AJ, Oeljeklaus S, Niemann M, Warscheid B, et al. Mitochondrial preprotein translocase of trypanosomatids has a bacterial origin. Curr Biol. 2011; 21: 1738–1743. doi: 10.1016/j.cub.2011.08.060 PMID: 22000100

55. Schöneck R, Billaut-Mulot O, Numrich P, Ouaissi MA, Krauth-Siegel RL. Cloning, sequencing and functional expression of dihydrolipoamide dehydrogenase from the human pathogen Trypanosoma cruzi. Eur J Biochem. 1997; 243: 739–747. PMID: 9057840

56. Kohl L, Sherwin T, Gull K. Assembly of the paraflagellar rod and the flagellum attachment zone complex during the Trypanosoma brucei cell cycle. J Eukaryot Microbiol. 1999; 46: 105–109. PMID: 10361731

57. Klein KG, Olson CL, Engman DM. Mitochondrial heat shock protein 70 is distributed throughout the mitochondrion in a dyskinetoplastic mutant of Trypanosoma brucei. Mol Biochem Parasitol. 1995; 70: 207–209. PMID: 7637705

58. Nilsson D, Gunasekera K, Mani J, Osteras M, Farinelli L, Baerlocher L, et al. Spliced leader trapping reveals widespread alternative splicing patterns in the highly dynamic transcriptome of Trypanosoma brucei. PLoS Pathog. 2010; 6: e1001037. doi: 10.1371/journal.ppat.1001037 PMID: 20700444
